# 5-azacytosine induces cytotoxicity via 5-methylcytosine depletion on chromatin-associated RNA in leukemia

**DOI:** 10.1016/j.molcel.2026.08.018

**Published:** 2026-09-15

**Authors:** Boyang Gao, Ying Li, Xinyuan Ma, Long Zhao, Zhongyu Zou, Juyeong Hong, Junhong Xiang, Xiaoyang Dou, Feng-Chun Yang, Mingjiang Xu, Chuan He

**Affiliations:** 1 Department of Molecular Genetics and Cell Biology, The University of Chicago, Chicago, IL 60637, USA; 2 Howard Hughes Medical Institute, The University of Chicago, Chicago, IL 60637, USA; 3 Department of Molecular Medicine, University of Texas Health Science Center at San Antonio, San Antonio, TX 78229, USA; 4 Department of Chemistry, The University of Chicago, Chicago, IL 60637, USA; 5 Department of Cell Systems & Anatomy, University of Texas Health Science Center at San Antonio, San Antonio, TX 78229, USA; 6 May’s Cancer Center, University of Texas Health Science Center at San Antonio, San Antonio, TX, USA; 7 Present address: Independent Research Groups, Max Planck Institute of Immunobiology and Epigenetics, Freiburg 79108, Germany; 8 These authors contributed equally; 9 Lead contact

## Abstract

5-azacytidine (5-azaC) is a DNA hypomethylating agent clinically used in myeloid malignancies. As a ribonucleoside analogue, 5-azaC incorporates more readily into nascent RNA than DNA. Here, we demonstrate that RNA 5-methylcytosine (m^5^C) depletion by 5-azaC treatment, particularly at early time points, is sufficient to induce leukemia cell death. In contrast to its DNA demethylation function, the RNA-dependent effects of 5-azaC lead to transcriptional repression, disrupting genes involved in cell-cycle regulation and DNA repair. Mechanistically, depletion of m^5^C by 5-azaC in chromatin-associated RNA (caRNA) disrupts the MBD6-mediated H2AK119ub deubiquitination. This also impairs SRSF2 recruitment and the downstream H3K27ac deposition by p300. Consistently, loss of the caRNA methyltransferase NSUN2 caused prolonged cell cycle, defective DNA repair, and shifted hematopoietic lineage commitment toward erythropoiesis, mirroring the effects of 5-azaC treatment. Our findings highlight the transcriptional repression by 5-azaC through depleting caRNA m^5^C, providing additional insights into the mechanism of action for 5-azaC.

## INTRODUCTION

5-azaC is clinically used for high-risk myelodysplastic syndromes (MDSs)^1^ and acute myeloid leukemia (AML)^2–4^, yet frequent resistance limits its efficacy and points to an incomplete understanding of its mechanism of action. Although 5-azaC is primarily studied as a DNA hypomethylating agent (HMA), DNA demethylation alone cannot explain the differentially expressed genes (DEGs) observed following 5-azaC treatment^5–9^. Meanwhile, only 10%-20% of 5-azaC incorporates into DNA and 80%-90% into RNA^10^ (Figure S1A), whereby it covalently inhibits both DNA and RNA 5-methylcytosine (m^5^C) methyltransferases^11–15^. Supporting a potential RNA-dependent role, the DNA-exclusive deoxy form of 5-azaC, decitabine, exhibits lower clinical response rates than 5-azaC^16–21^. Notably, sensitivity to 5-azaC has been shown to be associated with expression levels and regulatory roles of RNA m^5^C methyltransferases^22^. These findings indicate an underappreciated role of RNA m^5^C depletion in driving 5-azaC cytotoxicity, yet detailed mechanisms are still lacking.

RNA m^5^C regulates multiple cellular processes^23,24^. It influences rRNA biogenesis^25^ and tRNA stability^26^, both contributing to translational regulation. In mRNA, m^5^C may affect splicing^27^, nuclear export^28^, and RNA stability^29,30^, likely mediated by the m^5^C-binding proteins SRSF2, ALYREF, or YBX1, respectively. We recently discovered a transcriptional regulatory role of m^5^C in caRNA^31^, through its recognition by MBD6^31^ and MBD5^32^, which then recruit the BAP1 complex (PR-DUB) for the removal of histone H2AK119ub to confer transcriptional activation. Oxidation of caRNA m^5^C by TET2 suppresses this gene activation pathway^31^ and is associated with leukemogenesis, suggesting a role in 5-azaC treatment.

In this study, we characterized the transcriptional impacts of 5-azaC treatment. Contrary to the HMA role, RNA-dependent effects caused transcriptional repression. By characterizing early time points of treatment, we uncovered the RNA-dependent pathways that disrupt cell-cycle regulation, attenuate DNA damage repair, and promote apoptosis, leading to leukemia cell death. Mechanistically, m^5^C depletion on caRNA reduces MBD6 binding and subsequent recruitment of the BAP1 deubiquitylase complex for histone H2AK119ub removal. In parallel, caRNA m^5^C depletion disrupts SRSF2 binding to chromatin, impairing H3K27ac deposition. The RNA-dependent effects by 5-azaC on transcription and cytotoxicity can be recapitulated through depletion of m^5^C methyltransferase NSUN2. *Nsun2* KO in mice led to a shift in hematopoietic commitment toward the erythroid lineage. Finally, 5-azaC sensitivity screen in leukemia cell lines identified TET2 and IKZF1 impairment that can enhance 5-azaC sensitivity. These findings highlight the RNA-dependent pathways in 5-azaC treatment, and provide new insights into efficacy prediction and novel therapy development based on 5-azaC.

## RESULTS

### 5-azaC induces RNA m^5^C depletion, transcriptional repression and leukemia cell death at early time points

To probe the overall transcriptional effects by 5-azaC^5–8^, we performed 5-ethynyl uridine (EU) labeling of nascent RNA following 5-azaC treatment at 3 μM, reflecting its peak clinical plasma concentration^33,34^. The same concentration was used throughout this study unless otherwise noted. Contrary to its DNA hypomethylation effect, 5-azaC led to transcriptional downregulation across leukemia cell lines and AML patient samples after 24 or 72 h of treatment (Figures 1A, 1B and S1B), indicating a previously unrecognized mechanism.

We hypothesized that 5-azaC represses transcription through RNA m^5^C depletion. This hypothesis is supported by our previous findings that RNA m^5^C induces H2AK119ub deubiquitylation^31^. To distinguish between RNA- and DNA-dependent impacts of 5-azaC, we reasoned that the effects of 5-azaC could be primarily RNA-dependent at early treatment stages, as its DNA effects require rate-limiting deoxygenation and passive dilution of existing DNA methylation (Figure S1A). We measured RNA m^5^C and DNA 5mC levels using ultra-high-performance liquid chromatography-tandem mass spectrometry (UHPLC-MS/MS), and observed decreased m^5^C levels on tRNA and caRNA starting at 8 h, with caRNA showing a greater decrease (Figure 1C). The decrease in tRNA m^5^C is consistent with previous reports showing inhibition of TRDMT1-targeted methylation on tRNA by 5-azaC^35^. In contrast, 5mC levels in genomic DNA were not significantly changed within 24 h (Figure 1C). We therefore conclude that 5-azaC treatment between 8 and 24 h predominantly reflects its RNA m^5^C-dependent functions.

The 24-h 5-azaC-induced EU incorporation decrease (Figures 1A, 1B and S1B) supports RNA-dependent transcriptional repression. We also performed nascent RNA-seq^31^ (Figures S1C and D), which captures newly synthesized RNA through biotin labeling of incorporated EU. The levels of nascent RNA were normalized to viable cell numbers by adding EU-labeled Drosophila spike-in. Significant transcriptional downregulation emerged at 8 h (Figures S1C and S1D), coinciding with the initial decrease in the caRNA m^5^C level (Figure 1C). Importantly, minimal transcriptional changes were observed within the first 4 h of 5-azaC treatment, arguing against global transcriptional repression resulting from inhibition of nucleic acid synthesis, which has been reported within 4-h treatment window at much higher 5-azaC concentrations^10^. Together, these findings support a model where 5-azaC induces transcriptional repression through RNA m^5^C depletion.

5-azaC inactivates methyltransferases by forming cross-linking adducts when it is incorporated into nucleic acids^11,12,36^. We detected the cross-linking of NSUN2, the RNA m^5^C methyltransferase that has been reported to impact transcriptional activity^31^. By performing aza-IP after 5-azaC treatment in MOLM13, we observed cross-linking between RNA and NSUN2 that is only slightly detected at 4 h, but peaks after 8 h of treatment (Figure S1E). Considering that the cross-linking precedes RNA m^5^C depletion, our finding is consistent with the initial decrease of RNA m^5^C (Figure 1C) and transcriptional activity (Figures S1C and S1D) at 8-h treatment, supporting the dynamics of 5-azaC-mediated impacts we proposed.

Interestingly, despite the anticipated transcriptional upregulation due to DNA demethylation at later time points, RNA-dependent effects appeared to still dominate, and transcriptional levels remained decreased after 72 h of treatment (Figures 1A, 1B and S1B). The transcriptional level remained downregulated for 5 days of 5-azaC treatment, before returning to control levels on Day 8 (Figure S1F). This aligns with previous reports of inconsistencies between differential gene expression and DNA methylation changes following 5-azaC treatment^5–8^, highlighting the critical role of RNA-dependent mechanisms in 5-azaC-mediated gene expression regulation.

### RNA-dependent effects of 5-azaC induce leukemia cell death

Although RNA-dependent pathways were proposed previously for 5-azaC^14,15,22,35,37^, it remains unclear whether these pathways could contribute to leukemia cell death. We show that 5-azaC induces significant proliferation inhibition within 24 h of treatment in multiple leukemia cell lines, whereas decitabine had little impact within the same treatment window (Figure 1D). These results suggest that the RNA-dependent pathways alone can cause leukemia cell death. Notably, MOLM13 and SKM1, both derived from AML patients with prior MDS progression, were more susceptible to 5-azaC treatment at early time points (Figure 1D), contrary to THP1 and K562.

It has been reported that 5-azaC could induce apoptosis^38^. We hypothesized that the RNA-dependent cytotoxicity of 5-azaC is also caused by apoptotic responses. The terminal deoxynucleotidyl transferase dUTP nick end labeling (TUNEL) assay detected apoptosis after treatment with 5-azaC but not decitabine (Figure 1E), supporting our hypothesis.

To understand how transcriptional dysregulation contributes to the apoptotic effects of 5-azaC, we performed RNA-seq following 24-h 5-azaC treatment in MOLM13 and SKM1 cells (Figures S2A and S2B). Gene set enrichment analysis (GSEA) revealed upregulation of apoptotic genes (Figure 1F), consistent with our findings by TUNEL assay. The DEGs of both cell lines enriched in pathways associated with cancer cell survival. Gene ontology (GO) analysis of biological processes showed that downregulated DEGs were involved in cell division, DNA repair, and RNA processing, while upregulated DEGs were linked to the inflammatory response (Figures S2C and S2D).

As previously established, defective cell-cycle progression induces apoptosis. Multiple pathways related to cell division were downregulated after 5-azaC treatment, including mitotic cell cycle, DNA replication, and chromosome segregation (Figures S2C and S2D). Gene set enrichment analysis (GSEA) revealed downregulation of cell cycle-related genes from RNA-seq (Figure 1G) and nascent RNA-seq analysis (Figures S2E and S2F). We further confirmed alterations in cell-cycle progression by 5-ethynyl-2′-deoxyuridine (EdU) labeling and Hoechst staining to distinguish G0/G1-, S- and G2/M-phase cells. A 16-h treatment with 5-azaC significantly decreased the S-phase cell population in MOLM13, SKM1, and an AML patient sample (Figures 1H, S2G and S2H). These observations suggest that 5-azaC treatment at early time points prolongs the leukemia cell cycle, leading to proliferation inhibition that may also contribute to apoptosis.

In parallel, apoptosis also arises from unsuccessful DNA repair, and DNA damage response inhibitors have been used in cancer therapy to enhance cell death^39^. GO analysis of RNA-seq revealed downregulation of DNA damage repair genes 24 h after 5-azaC treatment (Figures S2C and S2D). GSEA showed a collective downregulation of DNA repair pathway genes at both the RNA levels (Figure 1I) and transcriptional activities (Figures S2I and S2J). Immunoblotting further confirmed the downregulation of central checkpoint proteins, including CHK1 and RAD51 (Figure 1J and K). Functionally, we validated that UVC irradiation elicited diminished γH2A.X signaling following 5-azaC treatment (Figure 1L). UVC-induced unscheduled DNA synthesis (UDS), assessed via EdU incorporation with exclusion of S-phase cells, demonstrated impaired DNA repair activity after a 16-h exposure to 5-azaC, but not to decitabine (Figures 1M and S2K). These findings collectively uncover that the early effects of 5-azaC disrupt the DNA damage response in leukemic cells. The accumulation of DNA lesions may further impede cell-cycle progression, causing apoptotic response observed with 5-azaC treatment.

One of the DNA repair pathways, nucleotide excision repair (NER), is mediated by either global genome repair or transcription-coupled repair (TCR), with the latter dependent on transcriptional activity^40^. We reasoned that the overall transcriptional downregulation caused by 5-azaC might directly contribute to the impaired DNA damage response in addition to its gene expression regulation. Compared with controls, UDS after UVC irradiation was less susceptible to transcription inhibition in 5-azaC-treated cells (Figures S2L and S2M), implying a direct role of transcriptional suppression on the early DNA damage repair defects induced by 5-azaC.

To further explore the RNA-dependent effect of 5-azaC under physiological conditions, we performed a 24-h 5-azaC treatment on the Lin^−^c-KIT^+^ (LK) cells purified from mouse bone marrow (BM). GSEA analysis revealed similar downregulation of cell cycle and DNA repair genes (Figures 1N and 1O), suggesting a physiologically relevant mechanism.

Intriguingly, 5-azaC has been combined with the BCL2 inhibitor venetoclax for improved response in AML treatment^41,42^. We observed enhanced efficacy of proliferation inhibition for MOLM13 cells within 24 h of combined 5-azaC and venetoclax treatment, whereas no synergistic effect was observed for decitabine and venetoclax within the same time window (Figure S2N). These results support RNA-dependent cytotoxicity of 5-azaC in leukemic cells at early time points, and this effect can synergize with pro-apoptotic agents.

### 5-azaC induces transcriptional repression through the m^5^C-dependent H2AK119ub regulation

To explore how 5-azaC represses transcription, we reasoned that transcription is more likely to be regulated by m^5^C on caRNA rather than other RNA species. Our previous findings also showed that H2AK119ub removal by m^5^C is mediated through caRNA^31^. We first identified caRNA m^5^C sites using methylated RNA immunoprecipitation sequencing (MeRIP-seq) as previously reported^31^. caRNA m^5^C was reduced after a 16-h 5-azaC treatment in MOLM13 (Figure S3A), with 7,095 downregulated and 4,567 upregulated sites out of 21,158 peaks identified from control and treated samples. Notably, repetitive elements accounted for 62.6% of input reads and 88.27% of MeRIP-enriched reads (Figure S3C), highlighting repeat RNA as a crucial group of regulatory caRNA with m^5^C. Similarly, caRNA m^5^C MeRIP in SKM1 cells detected significant caRNA m^5^C downregulation after 5-azaC treatment (Figure S3B) and enrichment of repeat RNA (Figure S3D). Further analysis identified long terminal repeat (LTR) elements as the most modified repeat RNA (Figure S3E), consistent with our previous findings^31^, while some subfamilies of long interspersed nuclear elements (LINEs), short interspersed nuclear elements (SINEs), and DNA transposons were also m^5^C modified. 5-azaC treatment caused reduced m^5^C levels among most repeat RNAs (Figure S3F), consistent with the reduced overall caRNA m^5^C level measured with UHPLC-MS/MS (Figure 1C). RNA modifications on repeat RNA have emerged as key elements in the regulation of local chromatin state and transcription^31,43–45^, and caRNA m^5^C has been associated with maintaining an open chromatin state^31^. Therefore, the 5-azaC-induced m^5^C downregulation may drive the observed transcriptional repression. Indeed, 5-azaC treatment caused the downregulation of caRNA repeat subfamilies bearing m^5^C, particularly in the LTR family (Figure S3G). Furthermore, genes adjacent to m^5^C sites exhibited greater downregulation than those farther away (Figure S3H), reinforcing the link between m^5^C loss and transcriptional repression of local chromatin regions.

Consistent with our previously identified m^5^C/MBD6/PR-DUB/H2AK119ub axis^31^, a 16-h 5-azaC treatment caused elevated H2AK119ub (Figures 2A and S4A). H2AK119ub CUT&Tag analysis demonstrated elevated H2AK119ub levels near genomic regions of m^5^C-modified caRNA (Figures 2B and 2C). Consistent with the previously suggested role of LTRs in H2AK119ub regulation^31^, we observed the most significant upregulation of H2AK119ub at specific LTR subfamilies (Figure 2D). This upregulation was detected across gene loci and was particularly evident at transcription start sites (TSSs) (Figure S4B). Particularly, we observed increased promoter H2AK119ub at genes related to cell cycles and DNA damage repair pathways (Figure S4C). Similar upregulation of H2AK119ub was observed in 5-azaC-treated LK cells (Figure 2E). We further performed MBD6 Cross-linking immunoprecipitation (CLIP) followed by high-throughput sequencing (CLIP-seq) with sonication to release chromatin-bound fraction, which revealed 5-azaC-induced decreased MBD6 binding at sites with reduced m^5^C intensity in MeRIP-seq (Figures 2B and S4D), consistent with the role of MBD6 as an m^5^C-binding protein involved in H2AK119ub regulation.

To further validate the role of RNA m^5^C in this regulation, we depleted NSUN2, the primary methyltransferase responsible for installing m^5^C on caRNA^31^. *NSUN2* knockdown (KD) resulted in H2AK119ub upregulation (Figure 2F), supporting a causal role for m^5^C in this regulation. Notably, depletion of other m^5^C methyltransferases, including NSUN5, NOP2, NSUN6, NSUN7, and TRDMT1, did not induce similar H2AK119ub upregulation (Figures S4E and S4F). Furthermore, depletion of the DNA 5mC methyltransferase DNMT1 led to a reduction in H2AK119ub levels (Figure S4F), reinforcing that the early time-point effect of 5-azaC on H2AK119ub goes through the RNA m^5^C-dependent transcriptional repression rather than DNA hypomethylation.

MBD6 depletion has been shown to rescue the differentiation delay of *Tet2* deleted hematopoietic stem and progenitor cells (HSPCs) and inhibit leukemogenesis in *TET2* mutant leukemic cells, implying a potential role for MBD6 inhibition in leukemia treatment^31^. Consistently, *MBD6* KD sensitized MOLM13 cells to 5-azaC treatment (Figures 2G and S4G), indicating that combining 5-azaC with an MBD6 inhibitor could be a promising therapeutic approach for treating leukemia.

Overall, our findings support that 5-azaC represses transcription through caRNA m^5^C depletion that modulates transcription via the m^5^C/MBD6/PR-DUB/H2AK119ub axis.

### 5-azaC induces transcriptional repression through downregulation of H3K27ac

It has been reported that inhibitors of CBP/p300 exhibited a synergistic effect with 5-azaC but not with decitabine^46^, suggesting a link between the RNA m^5^C-dependent function of 5-azaC and H3K27ac regulation. We indeed observed overall decreased H3K27ac levels in MOLM13, SKM1 and AML patient-derived cell line 2017-129 after a 16-h 5-azaC treatment (Figures 3A and S5A), suggesting its role in m^5^C-dependent transcriptional repression. The reduction of H3K27ac persisted at later time points (Figures S5A and S5B), aligning with the predominant transcriptional repression effect of 5-azaC over DNA hypomethylation (Figures 1A, 1B and S1B). H3K27ac CUT&Tag analysis confirmed a global downregulation of H3K27ac (Figures 3B and S5C), particularly affecting genes related to cell cycles and DNA damage repair (Figures 3C and S5D). To elucidate the relationship between H3K27ac downregulation and transcriptional repression, we grouped genes into four quantiles based on the adjacent H3K27ac peak changes, with Q1 representing the most downregulated genes and Q4 the most upregulated. A comparison with nascent RNA-seq data revealed a correlation between H3K27ac loss and transcriptional repression in downregulated genes (Figures 3D and S5E). Most H3K27ac downregulation at repetitive elements occurred in LTRs and LINEs (Figures S5F and S5G). Decreased H3K27ac was observed at genomic loci corresponding to m^5^C-modified sites (Figures S5H and S5I), supporting RNA m^5^C-dependent H3K27ac downregulation by 5-azaC treatment.

NSUN2 depletion caused H3K27ac reduction by immunoblotting (Figures 3E and S5J), supporting the causal role of m^5^C. Among other m^5^C methyltransferases, we observed a subtle decrease of H3K27ac following *NSUN7* KD (Figure S5J), consistent with its reported role in eRNA m^5^C modification^47^. Depleting NSUN5, NOP2, NSUN6, or TRDMT1 did not induce H3K27ac reduction, while depletion of DNMT1 resulted in H3K27ac upregulation. The catalytic activity of NSUN2 is responsible for the regulation, as revealed by H3K27ac downregulation of cells treated with NSUN2 inhibitor DO-26B^48^ (Figure 3F). It is worth noting that DO-26B treatment caused a more pronounced reduction of H3K27ac compared to NSUN2 depletion, likely due to leakage from the Tet-on promoter driving sh*NSUN2* expression, as well as the longer induction period of NSUN2 depletion that may allow partial recovery of H3K27ac. To explore the genomic effects of NSUN2 depletion, we performed H3K27ac CUT&Tag in SKM1 with NSUN2 depletion, which revealed H3K27ac downregulation at m^5^C sites (Figure S5K), further supporting the causal relationship between m^5^C and H3K27ac.

### 5-azaC downregulates H3K27ac through suppressing SRSF2-mediated p300 recruitment

To elucidate the underlying mechanism connecting caRNA m^5^C to H3K27ac, we examined the m^5^C-binding protein SRSF2^27^, which has been reported to associate with the chromatin fraction and regulate transcription^49^. We validated its association with chromatin (Figure S6A), and detected H3K27ac reduction upon SRSF2 depletion (Figures 4A, S6B and S6C). Given that SKM1 cells exhibited remarkably higher SRSF2 expression than MOLM13 cells (Figure S6D), we selected SKM1 to explore H3K27ac regulation. Previous studies have shown that SRSF2 associates with p300, thereby influencing H3K27ac deposition^50,51^. Consistently, immunofluorescence analysis revealed colocalization of SRSF2 and p300 (Figures 4B and 4C). Proximity ligation assay (PLA) confirmed their interaction (Figure 4D), supporting that SRSF2 recruits p300 to regulate H3K27ac deposition.

Based on these observations, we hypothesized that 5-azaC disrupts m^5^C-guided genomic binding of SRSF2, thereby impairing p300 localization and H3K27ac installation. Due to the inability of commercial SRSF2 antibodies to efficiently immunoprecipitate SRSF2 (Figure S6E), we overexpressed SRSF2-Myc in K562 cells, and performed CLIP with end ligation of pCp-AF488 fluorophore. After 16 h of 5-azaC treatment, SRSF2 exhibited reduced RNA binding (Figure S6F). Given that SRSF2 binding to chromatin is known to be RNA-dependent^49^, we speculated that the impaired RNA binding of SRSF2 leads to reduced chromatin binding of SRSF2 and subsequent loss of H3K27ac deposition. SRSF2 CUT&Tag after 16 h of 5-azaC treatment revealed a marked reduction in SRSF2 chromatin binding, correlating with decreased H3K27ac (Figures 4E and 4G). SRSF2 binding was highly enriched at promoters of cell cycle and DNA repair genes, yet abolished following 5-azaC treatment (Figure S6G). This is consistent with the recent findings that genes involved in DNA damage repair are particularly sensitive to transcriptional regulation by SRSF2^52^. Furthermore, SRSF2 binding was downregulated at chromatin regions adjacent to m^5^C sites (Figure S6H). Our findings therefore support impaired SRSF2 recruitment and H3K27ac deposition caused by 5-azaC.

To establish a direct link between m^5^C and SRSF2 chromatin binding, we performed SRSF2 CUT&Tag after NSUN2 depletion in SKM1 cells. SRSF2 chromatin binding downregulated by 5-azaC was also reduced by NSUN2 depletion (Figures 4F and 4G). The reduction was most pronounced at TSS (Figure S6I), particularly for genes involved in cell cycle and DNA damage repair pathways (Figure S6J), aligning with early transcriptional changes induced by 5-azaC.

In summary, we uncovered a parallel pathway of 5-azaC-induced transcriptional repression through the m^5^C/SRSF2/p300/H3K27ac axis, highlighting RNA m^5^C as the critical link in epigenetic regulation and transcriptional repression effect of 5-azaC.

### NSUN2 regulates leukemia cell proliferation and hematopoietic lineage commitment

Given that NSUN2 is the primary m^5^C methyltransferase responsible for the 5-azaC-mediated transcriptional repression, the cytotoxicity of 5-azaC should be recapitulated by NSUN2 depletion. Indeed, we observed proliferation inhibition after NSUN2 depletion in MOLM13 cells (Figure 5A). This aligns with previous observations in AML and other cancer cell types^53–55^. GSEA of RNA-seq datasets from *NSUN2* KD MOLM13 cells revealed downregulation of cell-cycle genes (Figure 5B). NSUN2 depletion led to a decreased S-phase population in MOLM13 and SKM1 cells (Figure 5C and S7A), indicating prolonged cell-cycle progression. Additionally, GSEA also revealed downregulation of DNA damage repair genes (Figure 5D). We validated the reduced levels of CHK1 and RAD51 through immunoblotting (Figure 5E). NSUN2 depletion caused diminished γH2A.X signaling (Figure 5F) and defective DNA repair activity upon UVC irradiation (Figure 5G and S7B). Overall, the cytotoxicity of 5-azaC could be partially rescued by NSUN2 overexpression (Figure S7C), reinforcing the causal role of RNA m^5^C.

We next evaluated the long-term impacts of NSUN2 inhibition by 5-azaC through investigating *Nsun2* knockout (KO) in mice. MDSs are typically characterized by ineffective erythropoiesis^56^, and monocytosis has been associated with earlier transformation to AML^57^. The use of 5-azaC helps restore erythroid differentiation^58,59^. In 6-month-old *Nsun2* KO mice (Figure S7D), purified BM cells exhibited a reduced frequency of Lin^−^Sca1^+^c-Kit^+^ (LSK) cells (Figure 5H), indicative of impaired HSPC maintenance. We also observed decreased granulocyte-monocyte progenitor (GMP) cells and increased common myeloid progenitor (CMP) cells (Figure 5H). Additionally, *Nsun2* KO mice exhibited a higher population of Ter119^+^ erythroid cells in the spleen (Figure 5I), suggesting a shift toward erythropoiesis, consistent with the therapeutic effects of 5-azaC^58,59^. *In vitro* colony-forming unit cell (CFU-C) assays using *Nsun2* KO BM cells revealed decreased frequency and number of CFU-granulocyte/macrophage (CFU-GM) colonies, accompanied by an increase in burst-forming unit-erythroid (BFU-E) and CFU-granulocyte-erythrocyte-monocyte-megakaryocyte (CFU-GEMM) colonies (Figure 5J). CFU-GEMM colonies derived from *Nsun2* KO BM cells were larger in size (Figure 5K). The progeny of *Nsun2* KO CFU-C cultures exhibited a reduced LK population (Figure S7E) and an increased proportion of Ter119^+^ erythroid cells (Figure S7F), further supporting a shift toward erythropoiesis with impeded myelopoiesis after *Nsun2* deletion.

To further evaluate the impact of *Nsun2* loss on the repopulating capacity of HSPCs, we performed *in vivo* competitive repopulation assays. Total donor BM cells (CD45.2^+^) from wild-type (WT) or *Nsun2* KO mice were mixed with equal numbers of competitor BM cells (CD45.1^+^) and transplanted into lethally irradiated recipient mice (CD45.1^+^). While CD45.2^+^ donor cells from WT remained at ~50% in peripheral blood (PB), CD45.2^+^ cells from *Nsun2* KO BM progressively declined, reaching less than 10% at 32 weeks after transplantation (Figure S7G). Similar trends were observed in recipient BM 32 weeks after transplantation (Figure S7H). Furthermore, the *Nsun2* KO donor cells produced a significantly lower percentage of GMP cells in recipient BM (Figure S7I), leading to significantly fewer myeloid (Gr-1^+^/CD11b^+^) cells in PB and BM, as well as increased Ter119^+^ erythroid cells in BM (Figures S7J and S7K). These results suggest an essential role for NSUN2 in hematopoiesis maintenance and HSPC function *in vivo*, particularly in myeloid and erythroid lineage commitment and differentiation.

Given the established role of erythropoiesis in MDS^60^, we were particularly interested in the impact of NSUN2 on erythroid differentiation. *In vitro* erythroid differentiation using BM-derived HSPCs revealed enhanced erythroid differentiation upon *Nsun2* deficiency (Figures 5L, S7L, S7M, and S7N). Overall, our results demonstrate that NSUN2 depletion significantly promotes erythroid lineage commitment, effects consistent with those observed with 5-azaC treatment.

### Correlation of leukemia cell line mutations with 5-azaC sensitivity

Leukemia cell lines exhibit varying sensitivity to 5-azaC (Figure 1D). While the DNA-dependent cytotoxicity of 5-azaC has been extensively studied^61–65^, the factors influencing sensitivity to its RNA-dependent effect remain poorly understood. We screened 26 different leukemia cell lines for their response to 5-azaC at the early time point (Figure 6A). A 24-h decitabine treatment had little impact on proliferation, whereas 5-azaC led to varying levels of growth inhibition across different cell lines. We integrated publicly available mutation data and identified mutations enriched in 5-azaC-sensitive cells that are frequently observed in clinical MDS and AML samples. This includes *TET2*, which catalyzes the oxidation of RNA m^5^C^31^. It aligns with previous reports that MDS and AML patients harboring *TET2* mutations respond more effectively to 5-azaC treatment^59,61,66^, and the m^5^C/MBD6/PR-DUB/H2AK119ub regulatory axis we reported recently^31^ and in this study. Consistently, *TET2* KO THP1 cells exhibited increased sensitivity to 5-azaC, particularly at early time points (Figure 6B). Beyond *TET2*, mutations in *ASXL1*, a component of the PR-DUB complex, were also enriched in 5-azaC-sensitive cells.

In addition to RNA m^5^C-related pathways, we identified *IKZF1* as another potential class of functionally related mutations. While *IKZF1* mutations are more commonly observed in acute lymphoblastic leukemia (ALL)^67^, they have been identified in AML as an independent marker of adverse risk with unique clinical phenotypes^68^. Moreover, IKZF1 degraders have shown efficacy in eliminating *MLL1*-rearranged (*MLL*-r) AML cells^69^. In our proliferation assay, IKZF1 depletion sensitized K562 cells (Figures 6C and S8A) and NB4 cells (Figure 6D) to 5-azaC treatment. Cell-cycle analysis demonstrated a stronger decrease of S-phase population in IKZF1 depleted cells (Figures S8B and S8C). RNA-seq revealed that treatment with 5-azaC induced only subtle transcriptomic changes in K562 cells at 24 h, while IKZF1 depletion markedly amplified the transcriptional response (Figure 6E). Genes significantly up- or down-regulated by 5-azaC in IKZF1-depleted K562 cells (denoted as Sens-up and Sens-down, respectively) showed only mild up- and down-regulation in control cells (Figure 6F), but were notably down- and up-regulated after IKZF1 depletion (Figure 6G), suggesting that IKZF1 depletion may reprogram the transcriptomic landscape, rendering cells more reliant on Sens-down genes and more vulnerable to Sens-up gene activation, thereby increasing sensitivity to 5-azaC. IKZF1 ChIP-seq from the ENCODE: ENCSR948VFL^70,71^ revealed enrichment of IKZF1 binding at corresponding genomic loci of K562 m^5^C^31^ (Figure S8D), suggesting a functional interplay between IKZF1 and RNA m^5^C-mediated regulation.

To further investigate the relationship between gene expression levels and 5-azaC sensitivity, we applied the network inference algorithm NetBID2^72^ to model regulatory activities of frequently mutated genes in MDS and AML. We constructed a leukemia-specific gene regulatory network using SJARACNe algorithm, employing RNA-seq datasets of 72 myeloid-origin leukemia cell lines from the DepMap portal (DepMap: 24Q2).^73^ After overlaying this network with the five leukemia cell lines most sensitive to 5-azaC (SKM1, MOLM13, HEL, NOMO1, and OCI-AML3) and six most 5-azaC resistant ones (K562, NB4, TF1, SKNO1, THP1, and HL60), we assessed hub gene activity based on two criteria: 1) differential expression between sensitive and resistant cell lines; and 2) differential expression of target genes between the two groups (Figure S8E). This analysis identified three candidate genes related to 5-azaC sensitivity: *SRSF2*, *NPM1*, and *EZH2* (Figures S8F and S8G). The expression levels of these genes and their targets in leukemic cells correlate with 5-azaC sensitivity (Figure S8F). Positively regulated target genes of these candidate drivers are expressed at lower levels in sensitive cell lines, while negatively regulated targets are more highly expressed (Figure S8G). The identified association between the SRSF2 level and 5-azaC sensitivity supports the m^5^C/SRSF2/p300/H3K27ac axis we uncovered in this study. Additionally, *EZH2* mutations were enriched in 5-azaC-sensitive leukemic cells (Figure 6A). Functional validation revealed that *NPM1* KD moderately increased THP1 sensitivity to 5-azaC (Figure S8H), and pretreatment of HEL cells with EZH2 inhibitor tazemetostat led to a modest increase in 5-azaC sensitivity (Figure S8I). Overall, our leukemia cell line screen and network analysis identified genes associated with 5-azaC sensitivity, which may facilitate future therapeutic development and efficacy prediction.

## DISCUSSION

5-azaC incorporates into nascent RNA and DNA to covalently inhibit methyltransferases. While it was traditionally regarded as a DNA HMA, here we demonstrate that: 1) 5-azaC treatment causes RNA m^5^C hypomethylation within the first 24 h, and this effect is sufficient to drive cytotoxicity in leukemic cells, independent of DNA hypomethylation; 2) the RNA m^5^C hypomethylation explains the gene repression effect of 5-azaC; 3) we identify two pathways responsible for the observed gene repression by 5-azaC. Depletion of m^5^C in caRNA disrupts the MBD6-mediated H2AK119ub deubiquitylation, leading to globally elevated H2AK119ub levels. In parallel, it impairs SRSF2 binding to chromatin, reducing p300 recruitment for H3K27ac deposition; 4) NSUN2 is the primary target responsible for 5-azaC-induced gene repression, and our animal model studies demonstrate that Nsun2 depletion in HSPCs causes hematopoietic lineage commitment toward erythroid, reminiscent of that also observed in patients treated with 5-azaC^58,59^ ; and lastly 5) our leukemia cell line screen identified *TET2* or *IKZF1* depletion that can sensitize 5-azaC treatment, consistent with the RNA-dependent cytotoxicity of 5-azaC in leukemic cells. In summary, our findings highlight the transcriptional repression effect of 5-azaC through caRNA m^5^C depletion, providing insights into its mechanism of action that may facilitate future prediction of treatment efficacy and therapy developments based on 5-azaC.

Some patients benefited from 5-azaC treatment did not exhibit DNA demethylation^74,75^, and hypomethylated samples displayed differential gene expression patterns that could not be explained by DNA methylation changes^5–8^. By examining transcriptional dysregulation at early time points, we demonstrated that the NSUN2-mediated RNA methylation pathways are responsible for transcriptional repression observed with 5-azaC treatment. The RNA-dependent effect may be more transient due to the dynamic nature of RNA synthesis and degradation, in contrast to the heritable stability of DNA methylation. This potentially explains the necessity for continuous administration of 5-azaC in treatment^76^. The opposing effects of RNA- and DNA-dependent transcriptional regulation may amplify or attenuate each other depending on the genetic backgrounds of recipient individuals. For patient selectively respond to RNA methylation inhibition, it could be beneficial to develop RNA m^5^C-specific inhibitors for precise therapies.

We show that NSUN2 is the primary enzyme responsible for the caRNA demethylation and transcriptional repression caused by 5-azaC treatment. *Nsun2* KO mice revealed shifted hematopoietic lineage commitment toward erythropoiesis, consistent with the role of 5-azaC in restoring erythroid differentiation^58,59^. This study primarily focused on the caRNA-dependent transcriptional repression by 5-azaC, but its effects on other RNA species (mRNA, rRNA, and tRNA) should be explored in the future. A recently published paper reported that 5-azaC induces mRNA damage and activates the stress response^37^, providing a parallel RNA-dependent pathway regulated by 5-azaC. Additionally, it has long been known that 5-azaC depletes tRNA m^5^C. However, how this mechanism contributes to the cytotoxicity of 5-azaC remains to be determined. Given the relatively modest reduction in tRNA m^5^C and its maximal depletion at later treatment time point (48 hr, ~40% decrease; Figure 1C), we speculate that tRNA-dependent cytotoxic effects may become prominent at later stages, reflecting the long half-life of tRNA. The cytotoxicity, global transcriptional repression, and stress response observed at the early stage are most likely dominated by caRNA-dependent transcriptional regulation.

The heterogeneity of myeloid malignancies caused varying responses to 5-azaC treatment. A previous study comparing 5-azaC-sensitive and -resistant cell lines found differences in RNA m^5^C methyltransferase levels and their related transcriptional regulation^22^. Our leukemia cell line screen and gene network inference further revealed RNA m^5^C-related genes associated with 5-azaC sensitivity, including *TET2*, *ASXL1*, and *SRSF2*. Additionally, we identified EZH2, the methyltransferase for H3K27me3, which is closely linked to the regulation of H2AK119ub^77,78^ described above. In parallel, we revealed that IKZF1 depletion sensitizes leukemic cells to 5-azaC, suggesting IKZF1 as a potential new target to synergize with 5-azaC.

Recent clinical trials demonstrate HMAs, in combination with venetoclax, display higher therapeutic efficacy than HMAs alone^41,42^. Our findings revealed the contribution of RNA-dependent transcription suppression pathway to the enhanced efficacy of 5-azaC when combined with venetoclax. Although it has been suggested that 5-azaC and decitabine showed comparable efficacies when combined with venetoclax^79^, the mechanistic differences between 5-azaC and decitabine may point to new strategies to improve 5-azaC.

### Limitations of the study

The mechanistic findings of this study are limited to leukemia cell lines and patient-derived cell lines; therefore, these discoveries require further validation in clinical settings. The identified common mutations associated with 5-azaC sensitivity suggest involvement of these genes in relevant molecular pathways but do not provide direct guidance for predicting 5-azaC efficacy, as these conclusions are not yet supported by patient-derived data. Additionally, the observed hematopoietic lineage changes in *Nsun2* KO mice represent a correlation rather than definitive evidence that RNA-dependent effects of 5-azaC contribute to erythroid lineage conversion in MDS patients.

## RESOURCE AVAILABILITY

### Lead contact

Further information and requests for resources and reagents should be directed to and will be fulfilled by the lead contact, Chuan He (chuanhe@uchicago.edu).

### Materials availability

All the materials generated in this manuscript are available from the lead contact under a complete Materials Transfer Agreement.

## STAR★METHODS

### EXPERIMENTAL MODEL AND STUDY PARTICIPANT DETAILS

#### Cell culture and cell line construction

All of the human cell lines used are cultured at 37°C with 5% CO_2_. The following cell lines were kindly provided by the J. Chen laboratory^92^: MA9.3 (male), MA9.3ITD (male), MA9.3RAS (male), MA9.6 (male), MA9.6ITD (male), MA9.6RAS (male), KOPN1, SKNO1, KOCL69, JURKAT, KOCL50, TF1, HEL, and NB4. In human cell lines, K562, MOLM13, OCI-AML3, SKM1, NOMO1, CEM/C1, CCRF/CEM, HL60, THP1, CESS, ARH77, KOPN1, KOCL69, JURKAT, KOCL50, HEL, and NB4 were maintained in RPMI1640 medium supplemented with 10% FBS and 50 U/mL Penicillin-Streptomycin. KASUMI1 cells were cultured in RPMI1640 with 10% FBS and 50 U/mL P/S. TF1 cells were kept in RPMI1640 with 10% FBS, 50 U/mL P/S and 2 ng/mL human GM-CSF. SKNO1 cells were grown in RPMI1640 with 10% FBS, 50 U/mL P/S and 10 ng/mL human GM-CSF. MA9.3 and MA9.6 cells were cultured in IMDM supplemented with 20% FBS, 50 U/mL P/S, 10 ng/mL each of human cytokines SCF, TPO, FLT3L, IL-3, and IL-6. MA9.3ITD, MA9.3RAS, MA9.6ITD and MA9.6RAS were kept in IMDM with 20% FBS and 50 U/mL P/S. HEK293T cells are used for lentivirus production and were kept in DMEM (high glucose, pyruvate) with 10% FBS and 50 U/mL P/S. Mycoplasma contamination was routinely tested using qPCR every half a year. *Drosophila* S2 cells were kept in Schneider’s Drosophila Medium with 10% FBS and 50 U/mL P/S and cultured at 27°C without CO_2_ supply. The sex information of the commercial cell lines can be found on their commercial website.

#### Human primary AML patient samples

Human primary AML patient samples were collected at the time of diagnosis, relapse or remission after written informed consent at City of Hope Hospital or MD Anderson Cancer Center incongruence with the protocol approved by the institutional review board (IRB). Characteristics of the AML patients are provided in Table S3. The AML patient samples were cryopreserved in liquid nitrogen for further study. AML #2017-129, #2016-35 and #6303 cells were cultured in StemSpan^™^ SFEMII supplemented with 10ng/mL of human SCF, mouse TPO, mouse FLT3L, human IL-3, human IL-6, 35 nM of UM171, 500 nM of Stem regenin1 (SR1) and 50 U/mL of P/S.

#### Animals

The global *Nsun2* KO mouse model was obtained from Frye’s lab^93^. These mice used in this study were backcrossed for more than 6 generations with C57BL/6 mice and applied throughout this study, including both male and female. All animal studies were performed with the approval from the Institutional Animal Care and Use Committee (IACUC) at The University of Texas Health Science Center at San Antonio (UTHSCSA) and conducted in accordance with the institutional and national guidelines and regulations. 6-month-old mice were used for experiment unless otherwise specified.

### METHOD DETAILS

#### Cell line construction

Lentiviral transduction was performed using viral particles packaged with HEK293T cells with pCMV-VSV-G and pCMV-dR8.2 plasmids (Addgene 8454 and 8455). Viral particles were precipitated using TransDux MAX Lentivirus Transduction Reagent and used immediately following overnight precipitation.

Transfection of K562 cells was conducted using Lipofectamine^™^ LTX Reagent with PLUS^™^ Reagent, following the manufacturer’s recommended protocol for K562. Cells were cultured to reach a concentration of 0.8 million/mL, and suspended in 25 mL Opti-MEM^™^ I Reduced Serum Medium. A DNA mixture containing 30 μg plasmid and 30 μL PLUS^™^ Reagent in 3.75 mL Opti-MEM^™^ medium was incubated at room temperature (RT) for 10 mins. Subsequently, 120 μL LTX Reagent was added and incubated for 25 mins at RT before being introduced to the cell culture. Transfection was conducted for 8 hours before replacing the media with standard growth medium.

Nucleofection of HEL cells was performed using Lonza 4D-Nucleofector System with the SF cell line 4D-nucleofector kit according to the manufacturer’s protocol. Each reaction utilized 1 million cells freshly passaged at 0.2 million/mL the day before. Cells were electroporated using program DC102.

#### Cell proliferation assay and 5-azaC sensitivity screen

Cell lines expressing Dox-induced shRNA were treated with the indicated compounds following 5 days of Dox induction with replenishment every 24 hours. As an exception, proliferation of MOLM13 with NSUN2 depletion was measured from the first treatment of Dox. In continuous measurement of proliferation, cells were passaged every 2 days, and the cumulative proliferation was adjusted by accounting for passaging ratios during proliferation measurement. HEL cells subjected to gene overexpression were treated with drugs 24 hours post-nucleofection. All proliferation assays were initiated at a seeding density of 0.2 million/mL and maintained with drug treatments for the specified time course. Cell proliferation was quantified using CellTiter-Glo^®^ 2.0 Cell Viability Assay, according to the manufacturer’s protocol with modifications for enhanced lysis efficiency. Specifically, CellTiter-Glo^®^ 2.0 reagent was freshly diluted in RIPA buffer (25 mM Tris-HCl pH 7.6, 150 mM NaCl, 1% NP-40, 1% Na-deoxycholate, 0.1% SDS) at a 3:4 volume ratio. For each measurement, 100 μL of diluted reagent was added to 100 μL of cell culture, mixed vigorously for 2 mins using orbital shaking in “fast” mode, and incubated at RT for 10 mins. Luminescence was recorded using a BioTek Synergy HTX plate reader.

Proliferation responses to 5-azaC and decitabine across 26 leukemic cells lines were evaluated after 24-hour treatment across five concentrations. Proliferation profiles were clustered based on normalized luminescence signals across doses. The top 50 recurrent leukemia-associated mutations were selected for visualization, based on the number of mutated cases in leukemia reported by the curated set of non-redundant studies reported in cBioPortal^94–96^. Mutation annotations were retrieved from the DepMap database^73^ and supplemented using data from COSMIC^97^ and Cellosaurus^98^. Silent mutations were excluded from the final integrated mutation dataset.

#### *In vitro* colony-forming assay

Bone marrow (BM) cells were collected from wild-type or *Nsun2* KO mice and plated in triplicate in methylcellulose medium supplemented with 100 ng/mL mouse SCF, 10 ng/mL mouse IL-3, 50 ng/mL mouse TPO, 10 ng/mL mouse GM-CSF, 4 U/mL human EPO, and 50 ng/mL human interleukin-6. The colonies were imaged by STEMvision^™^ (STEMCELL Technologies) and scored on day 7. Colony cells were harvested and analyzed for expression of stem and progenitor markers and erythroid linage markers by flow cytometry.

#### Competitive repopulation assay

Competitive repopulation assay was performed to assess the effect of NSUN2 on the repopulating potential of HSPCs *in vivo*. 5 × 10^5^ total BM cells isolated from WT or *Nsun2* KO mice (CD45.2) were mixed with 5 × 10^5^ BoyJ (CD45.1) BM cells and transplanted into lethally irradiated (800 cGy) BoyJ recipients through the tail vein injection. The CD45.2/CD45.1 chimera in the PB were monitored monthly for 32 weeks. Recipients were sacrificed 32 weeks after transplantation to analyze the CD45.2/CD45.1 chimera in BM, as well as HSPCs and lineage cell distributions within CD45.1+ or CD45.2+ cell populations.

#### *In vitro* erythroid differentiation

The hematopoietic stem and progenitor Lin^−^c-Kit^+^ (LK) cell derived from BM were in suspension cultured for *in vitro* erythroid differentiation following protocols described previously^99,100^ with slight modifications. Briefly, LK cells were isolated from total BM cells of 6-9 months old mice using the Direct Lineage Cell Depletion Kit (Miltenyi Biotec, 130-110-470), and then the lineage-negative cells (Lin^−^) were sorted with c-Kit (CD117) MicroBeads (Miltenyi Biotec, 130-091-224) by magnetic-activated cell sorting with autoMACS^®^ Pro Separator (Miltenyi Biotec). LK cells were cultured in Iscove modified Dulbecco’s medium (IMDM) supplemented with 10% FBS, 100 ng/mL mSCF, 40 ng/mL mIGF-1 and 2 U/mL hEPO for expansion. After 4 days, cells were cultured in IMDM supplemented with 10% FBS, 100 ng/mL mSCF, 40 ng/mL mIGF-1 and 6 U/mL hEPO for erythroid differentiation. Erythroid cells were analyzed over time by FACS and Wright-Giemsa staining (Fisher Scientific, 23-123869).

#### Flow cytometry analysis

Flow cytometric analysis of mouse BM cells was performed as previously described^101^. Briefly, for HSPC cells, BM cells were stained with APC-anti-Lineage cocktail, PE-Cy^™^7-anti-Sca1, PE-anti-c-kit, Alexa Flour 700-anti-CD34, APC-Cy7-anti-CD16/32, BV421-anti-CD135. For analysis of myeloid lineage cells, BM cells were stained with PerCP-Cy^™^5.5-anti-Gr-1 and PE-anti-CD11b (Mac-1). APC-anti-Ter119 and PE-anti-CD71 were used for analyzing erythroid populations. APC-anti-B220 and APC-Cy7-anti-IgM were used for analyzing B lymphocytes. PE-anti-CD8a and PE-Cy^™^7-anti-CD4 were used for staining T lymphocytes.

#### Immunoblotting

Samples were collected with equal cell numbers and washed with PBS. For histone analysis, cells were lysed directly in 50 μL of 2x NuPAGE^™^ LDS sample buffer supplemented with β-mercaptoethanol (β-ME) per 1 million cells. To measure γH2A.X levels after UV damage, cells were exposed too 50 J/m^2^ UVC in PBS, then immediately incubated in complete medium for 5 mins prior to collection for immunoblotting. For analysis of non-chromatin-associated proteins, cells were lysed in 50 μL of RIPA buffer supplemented with 100x proteinase inhibitor cocktail per 1 million cells on ice for 15 mins. Lysates were then centrifuged at 12,000 x g for 15 mins at 4°C. The supernatant was mixed with NuPAGE^™^ LDS sample buffer containing β-ME to a final 1x concentration. Samples were denatured by heating at 95°C for 20 mins and sonicated until no longer viscous. The denatured protein samples were separated on 4–12% NuPAGE Bis-Tris gels (Invitrogen^™^, NP0335BOX) and transferred to 0.45 μm nitrocellulose membrane (Bio-Rad, 1620115) using a semi-dry transfer system at 25 V for 45 mins. Membranes were blocked in 5% BSA in PBSTat RT for 30 mins, followed by overnight incubation at 4°C with primary antibody diluted in PBST containing 3% BSA. After three 10-min washes in PBST, membranes were incubated with secondary antibody diluted in PBST containing 1% BSA for 40 mins at RT, washed three more times for 10 mins each, and developed using SuperSignal^™^ West Dura Extended Duration Substrate kit. Signals were visualized with the Invitrogen^™^ iBright imaging system. The SRSF2 antibody used for immunoblotting is Cat# ab204916 (Abcam).

#### Cellular fractionation and purification of caRNA

Cellular fractionation was performed based on previously described protocol^43^ with slight modifications. A total of 20 million cells were washed with PBS and pelleted by centrifugation at 500 x g. Cells were resuspended in 300 μL of ice-cold NP-40 lysis buffer (10 mM Tris-HCl pH 7.5, 150 mM NaCl, 0.05% NP40) by gently flicking the tube, avoiding pipetting. After 5 mins of incubation on ice, the suspension was layered onto ice-cold NP-40 lysis buffer containing 24% sucrose and centrifuged at 15,000 x g for 10 mins at 4°C. The supernatant was collected as the cytoplasmic fraction. The pellet was washed with ice-cold PBS and resuspended in 150 μL of ice-cold glycerol buffer (20 mM Tris-HCl pH 8.0, 75 mM NaCl, 0.5 mM EDTA, 50% glycerol) by flicking the tube. Next, 300 μL of ice-cold nuclei lysis buffer (10 mM HEPES pH 7.6, 7.5 mM MgCl2, 0.2 mM EDTA, 300 mM NaCl, 1M Urea, 1% NP40) was added to the suspension, which was then vortexed at maximum speed for 20 seconds, incubated on ice for 2 mins, and centrifuged at 15,000 x g for 2 mins at 4°C. The supernatant was collected as the nuclear fraction. The remaining pellet was washed with PBS and harvested as the chromatin fraction.

To isolate caRNA, the chromatin pellet was treated with 2 U TURBO DNase^™^ at 37 °C for 1 hour, followed by digestion with proteinase K at 55°C for 1 hour after supplementing 1% SDS to the buffer. TRIzol^™^ reagent was added to the mixture, which was then incubated at 50°C for 20 mins for resolution of pellets. Following TRIzol^™^ extraction with chloroform, the aqueous phase was collected, and RNA was purified using the RNA Clean & Concentrator-25 kit (RCC-25) including in-column DNase I digestion according to the manufacturer’s instructions.

#### UHPLC–MS/MS

70 ng of RNA was digested with nuclease P1 at 37 °C overnight, followed by treatment with 1 U shrimp alkaline phosphatase (rSAP) at 37 °C for 2 hours. Genomic DNA, purified from PureLink^™^ Genomic DNA Mini Kit, was digested with nuclease P1 at 37 °C overnight. This was followed by sequential digestion with 0.001 U phosphodiesterase I in 100 mM ammonium bicarbonate at 37 °C for 2 hours, and 1 U rSAP at 37 °C for 2 additional hours. Prior to analysis, all samples were diluted with LC-grade water and filtered through a 0.22 μm PVDF filter (Millipore, SLGVR04NL) prior to injection into a C18 reversed-phase column connected to a SCIEX 6500+ Triple Quadrupole Mass Spectrometer operating in positive electrospray ionization mode. Nucleosides were quantified based on retention time and specific nucleoside-to-base ion transitions (RNA: 268 → 136 for A; 258 → 126 for m^5^C; DNA: 252 → 136 for dA, 242 → 126 for 5mdC). Relative nucleoside levels were determined by peak area comparison within the same batch of reactions.

#### TUNEL assay

TUNEL assay was performed using the DeadEnd Fluorometric TUNEL System according to the manufacturer’s instructions, with the modification that the PBS wash buffer was supplemented with 0.5% BSA. Following rTdT labeling, the reaction was terminated using 20 mM EDTA containing 0.5% BSA in PBS to prevent cell clumping.

#### EU-based nascent RNA labeling analysis

Nascent RNA labeling for fluorescence detection was performed using the Click-iT^™^ RNA Alexa Fluor^™^ 488 Imaging Kit. Cells were incubated with 1 mM EU for 30 mins prior to harvesting. The procedure followed the manufacturer’s instructions, with modifications for flow cytometry analysis. At each wash step, cells were washed using 0.5% BSA in PBS to minimize cell clumping, and centrifuged at 3,000 x g for 3 mins at RT-except during the rinsing step, where the Click-iT^®^ reaction rinse buffer was used as instructed.

#### EdU-based cell cycle analysis and unscheduled DNA repair analysis

For cell cycle analysis, cells were incubated with 20 μM EdU for 1 hour, followed by a 30-min chase in complete media supplemented with 10 μM thymidine to dilute unincorporated EdU. Unscheduled DNA repair analysis was performed based on a published protocol^102^ with modifications. After treatment under designated conditions, cells were irradiated with 50 J/m^2^ 254 nm UVC in PBS, followed by immediate addition of complete media containing 20 μM EdU and 1 μM 5-Fluoro-2’-deoxyuridine. Cells were incubated for 1 hour under standard culture conditions, then washed twice with PBS and chased in complete media supplemented with 10 μM thymidine for 30 mins. At harvest, EdU detection was performed using Click-iT^™^ RNA Alexa Fluor^™^ 488 Imaging Kit, following a flow cytometry-adapted protocol as described for EU-based nascent RNA labeling. Post click chemistry, cells were stained with 2 μg/mL Hoechst 33342 for 15 mins at RT, washed, and analyzed by flow cytometry. Hoechst signal was quantified in linear scale. Cells with increased EdU incorporation and intermediate Hoechst intensity were classified as S phase, while 1N and 2N populations with basal level EdU intensity were designated as G0/G1 and G2/M phases, respectively. For unscheduled DNA repair analysis, only G0/G1 or G2/M populations were included in the quantification.

#### Immunofluorescence staining

Chamber slides were pre-coated with 0.1 mg/mL poly-D-lysine overnight to promote cell adherence. After harvest, cells were fixed with 4% paraformaldehyde (PFA) in PBS at RT for 10 mins, washed, and resuspended in PBS at a concentration of 1 million cells/mL. Cells were added to the pre-treated chambers and allowed to settle by gravity for 30 mins at RT without disturbance. Following sedimentation, excess PBS was gently removed, and cells were permeabilized by 0.5% Triton X-100 in PBS for 10 min, followed by PBS washes at RT. Cells were then blocked with 0.5% BSA in PBS or 20 min, and subsequently incubated with primary antibodies diluted in blocking buffer: anti-SRSF2 (ab11826) at ratio of 1:200 and anti-p300 at ratio of 1:400 for 1 hour at RT. After PBS washing, cells were incubated with secondary antibodies diluted 1:1000 in blocking buffer for 1 hour: goat anti-mouse IgG-AF 546 and goat anti-rabbit IgG-AF 647. Cells were then stained with 2 μg/mL Hoechst 33342 in PBS for 15 mins at RT. Following final PBS washes, ProLong^™^ Glass Antifade Mountant was applied to minimize photobleaching. Fluorescence images were acquired using a Leica SP8 laser scanning confocal microscope. The fluorescence intensity was measured by ImageJ.

#### Proximity ligation assay

Proximity ligation assay was conducted using the Duolink^®^ Proximity Ligation Assay kit. Cells were fixed with 4% PFA in PBS at RT for 10 mins, followed by gravity sedimentation at a concentration of 1 million cells/mL onto chamber slides pre-coated with 0.1 mg/mL poly-D-lysine overnight. After permeabilization with 0.5% Triton X-100 in PBS for 10 min, the assay was carried out according to the manufacturer’s protocol. Primary antibodies used were anti-SRSF2 (ab11826, 1:200) and anti-p300 (1:400). Following amplification, nuclei were stained with 2 μg/mL Hoechst 33342 for 15 mins, and slides were mounted using ProLong^™^ Glass Antifade Mountant. Fluorescence imaging was conducted using a Leica SP8 laser scanning confocal microscope.

#### Immunoprecipitation

Immunoprecipitation was performed using 50 μL of Dynabeads^™^ Protein G or Dynabeads^™^ Protein A, washed with lysis buffer (50 mM Tris-HCl pH 7.5, 100 mM NaCl, 1% NP-40 substitute, 0.5% sodium deoxycholate, 100 × proteinase inhibitor cocktail). Beads were conjugated with SRSF2 antibodies-20 μL of the one from Abcam (ab204916), 5 μg of the one from Proteintech or 5 μL of the one from Sigma-Aldrich-and Rabbit IgG or Mouse (G3A1) mAb IgG1 Isotype Control as controls. Protein G beads were used for mouse antibodies and protein A beads were used for rabbit antibodies. 20 million SKM1 cells per sample were lysed in 800 μL lysis buffer at 4 °C with end-to-end rotation for 30 mins. Lysates were centrifuged at 15,000 x g for 15 min at 4 °C, and the supernatant was collected. 40 μL of lysate was reserved as input, and the remainder was incubated with the antibody-conjugated beads at 4°C for 4 hours. Beads were washed three times with wash buffer (50 mM Tris-HCl pH 7.5, 300 mM KCl, 0.05% NP-40 substitute, 1000 × proteinase inhibitor cocktail). washed beads were suspended in 50 μL of 1x Laemmli sample buffer supplemented with β-ME and heated at 95 °C for 10 minutes. The input sample was mixed with 4x Laemmli sample buffer supplemented with β-ME to a final 1x concentration and heated the same. 5 μL of input and 20 μL of eluted sample were loaded for immunoblotting.

#### Aza-IP

Aza-IP was performed according to published protocol with slight modifications^103^. 8 x 10^7^ MOLM13 cells per sample were treated with 3 μM 5-azaC for 0, 4, 8 or 16 hours, followed by cell lysis with Pierce^™^ IP lysis buffer on ice for 5 min. The Cell lysate supernatant was harvest by centrifugation at 12,000 x g for 10 min at 4 °C, and incubated with 100 μL of Dynabeads^™^ Protein G pre-coated with 10 μL of the NSUN2 antibody or control IgG. After 4 hours of room temperature rotation, beads were washed with MeRIP buffer (10 mM Tris-HCl pH 7.5, 150 mM NaCl, 0.1% NP-40, 0.2 U/μL SUPERase·In^™^ RNase Inhibitor) and high salt buffer (10 mM Tris-HCl pH 7.5, 500 mM NaCl, 0.1% NP-40, 0.2 U/μL SUPERase·In^™^ RNase Inhibitor) twice respectively. The immunoprecipitated beads were digested with 0.2 U/μLRNaseT1 at room temperature for 15 min, and RNA was released by proteinase K digestion and purified by RNA Clean & Concentrator-25 kit. The same proportion of RNA samples were loaded on TapeStation for electroporation.

#### RNA-seq

RNA extraction was performed using TRIzol^™^ reagent following the designated treatment. The RNA was extracted with chloroform, and the aqueous phase was retained for RNA purification using RCC-25. In-column DNase I digestion was conducted to remove DNA. For studies involving gene depletion, KD efficiency was assessed by RT-qPCR. For RNA-seq after 5-azaC treatment, 2 μL of a 1/20,000 dilution of ERCC RNA spike-in mix was added to 10 ng of RNA. The RNA was then proceeded for library construction using the SMARTer^®^ Stranded Total RNA-Seq Kit v2 - Pico Input Mammalian. Next-generation sequencing was performed on Illumina NovaSeqX with pair-end 150 bp reads.

#### Nascent RNA-seq

MOLM13 and SKM1 cells were treated with 3 μM 5-azaC for 0, 4, 8 and 16 hours. Following treatment, 1 mM EU was added to the media for nascent RNA labeling. In parallel, *drosophila* cells were labeled similarly to serve as a spike-in control. Cells were harvested and purified using TRIzol^™^ reagent following the manufacturer’s protocol with isopropanol precipitation. Nascent RNA was enriched using the Click-iT^™^ Nascent RNA Capture Kit for gene expression analysis, following the manufacturer’s protocol with modifications. 4.5 μg of EU-labeled RNA was reacted with 2.2 μL biotin azide in a 25 μL click reaction and purified using RCC-25. 600 ng of RNA was mixed with *drosophila* spike-in at a 200:1 ratio and processed for nascent RNA enrichment. Streptavidin beads provided by the kit were washed with B&W buffer (5 mM Tris-HCl pH 7.5, 0.5 mM EDTA, 1M NaCl) three times, followed by solution A (100 mM NaOH, 50 mM NaCl) twice for 2 mins each. The beads were then washed with 100 mM NaCl at the same volume of solution A, followed by another round of washing with B&W buffer three times. Beads were suspended in the wash buffer 2 provided by the kit, and RNA was mixed with RNaseOUT in RNA binding buffer and denatured at 70°C for 5 mins before being added to the beads. After 30 mins of incubation at RT with end-to-end rotation, the beads were washed with low salt buffer (10 mM Tris-HCl pH 7.5, 50 mM NaCl, 0.1% NP-40, 0.2 U/μL SUPERase·In^™^ RNase Inhibitor), high salt buffer (10 mM Tris-HCl pH 7.5, 500 mM NaCl, 0.1% NP-40, 0.2 U/μL SUPERase·In^™^ RNase Inhibitor), and B&W buffer supplemented with 0.2 U/μL SUPERase·In^™^ RNase Inhibitor, washing five times each. The beads were then washed with the wash buffer 1 and wash buffer 2 supplied by the kit and eluted in 50 μL of 95% formamide supplemented with 2.5 mM EDTA by heating at 65°C for 5 mins, followed by 95°C for 5 mins. The supernatant was immediately harvested while still hot, and RNA was extracted using 1 mL TRIzol^™^ reagent. RNA was purified following the manufacturer’s protocol with isopropanol precipitation facilitated by glycogen. The library was constructed using the SMARTer^®^ Stranded Total RNA-Seq Kit v2 - Pico Input Mammalian, and sequencing was performed using the Illumina NovaSeqX with pair-end 50 bp reads.

#### m5C MeRIP-seq

m^5^C MeRIP-seq was performed as described in previous publication^31^. For MOLM13 cells, 0.02 million *drosophila* S2 cells were added to 20 million MOLM13 cells before proceeding with cellular fractionation to enrich caRNA, as outlined earlier. rRNA removal was carried out using RiboMinus Eukaryote System v2. 2 μg ribo-depleted caRNA was then used for m^5^C immunoprecipitation. RNA was fragmented using 1× NEBNext^®^ Magnesium RNA Fragmentation buffer at 94°C for 2 mins, and 5% of the sample was saved as input. The RNA was heated to 72°C for 2 mins and then placed immediately on ice for denaturation. Next, the RNA was added to protein G dynabeads conjugated with 2 μg anti-m^5^C antibody in 300 μL IP buffer (10 mM Tris-HCl pH 7.5, 150 mM NaCl, 0.05% Triton X-100, 1 mM spermidine, 0.2 U/μL SUPERase·In^™^ RNase Inhibitor), and incubated with end-to-end rotation at 4°C for 2 hours. The beads were washed three times with IP buffer and eluted with buffer RLT. Both input and MeRIP libraries were constructed using the SMARTer^®^ Stranded Total RNA-Seq Kit v2 - Pico Input Mammalian, and sequencing was performed with the Illumina NovaSeqX with pair-end 150 bp reads.

#### CUT&Tag

CUT&Tag was performed using the CUT&Tag-IT Assay Kit following the manufacturer’s instructions. 0.2 million cells were used per sample. For 5-azaC-treated CUT&Tag profiling of H2AK119ub and H3K27ac, 4,000 *drosophila* nuclei from CUT&Tag-IT^®^ Spike-In Control were added as a spike-in control. Primary antibodies used included 1 μL H2AK119ub antibody, 1 μL H3K27ac antibody (Cat# 39034) or 0.25 μL SRSF2 antibody (Cat# ab11826). Libraries were constructed according to the kit protocol and sequenced on the Illumina NovaSeqX platform with pair-end 150 bp reads.

#### CLIP-seq and CLIP with end ligation of pCp-AF488 fluorophore

For CLIP-seq, 100 million cells were used for each replicate. Following the designated treatment, cells were crosslinked in PBS using 4500 J/m^2^ UV at 254 nm. Cells were lysed in 200 μL iCLIP lysis buffer (50 mM Tris-HCl pH 7.5, 100 mM NaCl, 1% NP-40 substitute, 0.1% SDS, 0.5% sodium deoxycholate, 100× proteinase inhibitor cocktail and 0.2 U/μL SUPERase·In^™^ RNase Inhibitor) at 4°C for 15 mins with end-to-end rotation. SDS were then added to reach a final concentration of 1% for protein release from chromatin. Samples were sonicated (35% amplitude, 2s on / 4s off cycles, 1 min on ice) and diluted 10-fold with SDS-free iCLIP lysis buffer to reduce SDS back to 0.1%. After centrifugation at 15,000 x g for 30 mins at 4°C, the supernatant was harvested and treated with 0.2 U/μL RNase T1 at 22°C for 15 mins, then quenched on ice for 5 mins. 2% of the lysate was saved as input and digested with 10 U/μL RNase T1 at 22°C for 10 mins, followed by proteinase K digestion, RNA purification and end repair. The remaining lysate was incubated overnight at 4°C with protein A beads conjugated to 30 μL MBD6 antibodies (gifted from Prof. Lu Wang^104^) per sample. Beads were washed three times with CLIP wash buffer (50 mM Tris-HCl pH 7.5, 300 mM KCl, 0.05% NP-40, 1000× protein inhibitor cocktail and 0.2 U/μL SUPERase·In^™^ RNase Inhibitor), followed by digestion with 10 U/μL RNase T1 at 22°C for 10 mins. Beads were further washed for three times each with CLIP high salt buffer (50 mM Tris-HCl pH 7.5, 500 mM KCl, 0.05% NP-40, 100× protein inhibitor cocktail and 0.2 U/μL SUPERase·In^™^ RNase Inhibitor) and PNK buffer without DTT, and subjected to end-repair using T4 PNK. After proteinase K digestion, RNA was purified for library preparation. The input and CLIP libraries were constructed using the NEBNext^®^ Small RNA Library Prep Set and sequenced with Illumina NovaSeqX, pair-end 150 bp.

For CLIP with end ligation of pCp-AF488 fluorophore, K562 cells with SRSF2-myc overexpression after DMSO or 5-azaC treatment were counted to ensure the same number of cells used for experiment in each sample. Approximately 15 million cells were crosslinked by 4500 J/m^2^ UV at 254 nm in PBS. Samples were lysed with iCLIP lysis buffer, and lysates were cleared by centrifugation. Supernatants were treated with 0.2 U/μL RNase T1 at 22°C for 15 mins, quenched on ice, and incubated at 4°C for 4 hours with protein G beads conjugated to 4 μL Myc-tag antibodies. Following immunoprecipitation, beads were washed for three times with CLIP wash buffer, digested with 10 U/μL RNase T1 at 22°C for 8 mins, and further washed for three times each with CLIP high salt buffer and PNK buffer without DTT. Beads were subjected to RNA dephosphorylation using T4 PNK, then ligated overnight at 16°C in a 26 μL ligation reaction containing 2.4 μL T4 RNA ligase 1, 0.5 μL pCp-AF488, 0.3 μL 100 mM ATP, 0.4 μL SUPERase·In^™^ RNase Inhibitor, 0.6 μL 1% Tween-20, 0.9 μL DMSO and 9 μL 50% PEG8000. After ligation, samples were washed, denatured in 2x LDS sample buffer with β-ME at 70°C for 10 mins, and analyzed on a 4–12% NuPAGE Bis-Tris gel for fluorescence detection.

#### Data analysis of RNA-seq

Adapter trimming was performed using Cutadapt^82^, and reads were mapped to the hg38 human genome or mm10 mouse genome using HISAT2^83^. Gene-level read counts were quantified using featureCounts^85^ based on the hg38 refGene annotation. For differential gene expression analysis, DESeq2^80^ was applied to datasets without spike-in, while RUVSeq^81^ was used for normalization and analysis of datasets that included ERCC RNA spike-in mix. GSEA was conducted using the GSEA software suite^86,105^.

#### Data analysis of nascent RNA-seq

Adapter trimming was performed using Cutadapt^82^, and reads were mapped to hg38 human genome and dm6 *drosophila* genome by HISAT2^83^. Gene-level read counts were quantified using featureCounts^85^ based on the hg38 refGene annotation. For differential gene expression analysis, DESeq2^80^ was applied using the read counts mapped to *drosophila* chromosomes as spike-in normalization. GSEA was conducted using the GSEA software suite ^86,105^.

#### Data analysis of m^5^C MeRIP-seq

Adapter trimming was performed using Cutadapt^82^, and reads were deduplicated and aligned to the hg38 human genome and dm6 *drosophila* genome using HISAT2^83^. Gene-level read counts were quantified using featureCounts^85^ based on the hg38 refGene annotation. In parallel, reads mapping to repetitive elements were analyzed using Bowtie2^84^ followed by RepEnrich2^87^. input samples were analyzed as caRNA-seq for differential expression analysis of caRNA, covering both coding genes and repetitive elements. DESeq2^80^ was used for differential expression analysis. For samples from MOLM13 cells with *drosophila* spike-in, total read numbers mapped to each *drosophila* chromosome were used for normalization in DESeq2 analysis.

MeRIP peaks were identified using MACS3^88^, and read counts from both input and MeRIP libraries were quantified at peak regions to assess enrichment. Peaks were normalized to total mapped reads, or additionally normalized by *drosophila* spike-in for MOLM13 samples. Regions with MeRIP/input > 0 in either control or 5-azaC-treated samples were considered putative m^5^C sites. Sites with an enrichment fold change of 5-azaC control < 0.5 were defined as decreased m^5^C sites. Metaplots were generated using deepTools^89^. The IKZF1 ChIP data was downloaded from ENCODE experiment ENCSR948VFL.

#### Data analysis of CUT&Tag

Adapter trimming was performed using Cutadapt^82^, and reads were aligned to the hg38 human genome and dm6 *drosophila* genome using bowtie2^84^. Duplicate reads were removed using Picard MarkDuplicates^106^. For 5-azaC-treated histone modification profiling, signal normalization was performed based on the total number of reads mapped to *drosophila* genome. Peak calling was performed using MACS3^88^. For histone modifications, broad peaks were identified using the parameters “--broad --broad-cutoff 0.01” was used. Differential peak analysis was conducted using DiffBind^107^. For quantile-based analysis, peaks were annotated to the nearest RefSeq genes using Homer annotatePeaks.pl^90^, and assigned as their putative regulatory elements. Metaplots were generated with deepTools^89^.

#### Data analysis of CLIP-seq

Adapter trimming was performed using Cutadapt^82^, and reads were aligned to the hg38 human genome using HISAT2^83^. Duplicate reads were removed using Picard MarkDuplicates^106^. Metaplots were generated with deepTools^89^.

#### Inference of gene network

A gene regulatory network was inferred using SJARACNe^91^, based on gene expression profiles of all myeloid lineage leukemia cell lines available in the DepMap^73^ database. Candidate hub genes were selected from commonly mutated genes identified in MDS, AML and Myeloproliferative neoplasm, curated from non-redundant studies in cBioPortal^94–96^. Selection criteria included mutation frequency > 1%, mutation cases > 5 and total detected cases > 100. The network was further integrated with gene expression data from leukemia cell lines categorized as sensitive and resistant to 5-azaC, analyzed using NetBID2^72^.

### QUANTIFICATION AND STATISTICAL ANALYSIS

Data are plotted with error bars representing mean ± standard deviation. For each boxplot, the box represents 25%, 50% and 75% quantiles, while the whiskers extend to the most extreme data point that is within 1.5-fold IQR (interquantile range) of the box edge. Individual outliners outside this range are hidden. *P*-values indicated in the figures are unpaired, two-sided Student’s *t* test. An exception is the GSEA analysis, where the nominal *p*-values generated by the GSEA software are labeled in the figures.

## Supplementary Material

SupFile_5azaC

TableS1-oligo Table S1. Sequences of qPCR primers and shRNA, related to all figures.

TableS4-azaC-EU Table S4. Differential transcriptional rate of genes after early 5-azaC treatment, related to Figure 1.

TableS5-NSUN2KD-DE Table S5. Differential expression of genes after *NSUN2* depletion in MOLM13, related to Figure 5.

TableS3-AML patient Table S3. Genetic background of the AML patients in this study, related to Figure 1.

TableS2-24hrazaC_vs_0hr-DE Table S2. Differential expression of genes after 24-h 5-azaC treatment, related to Figure 1.

## Figures and Tables

**Figure 1.**
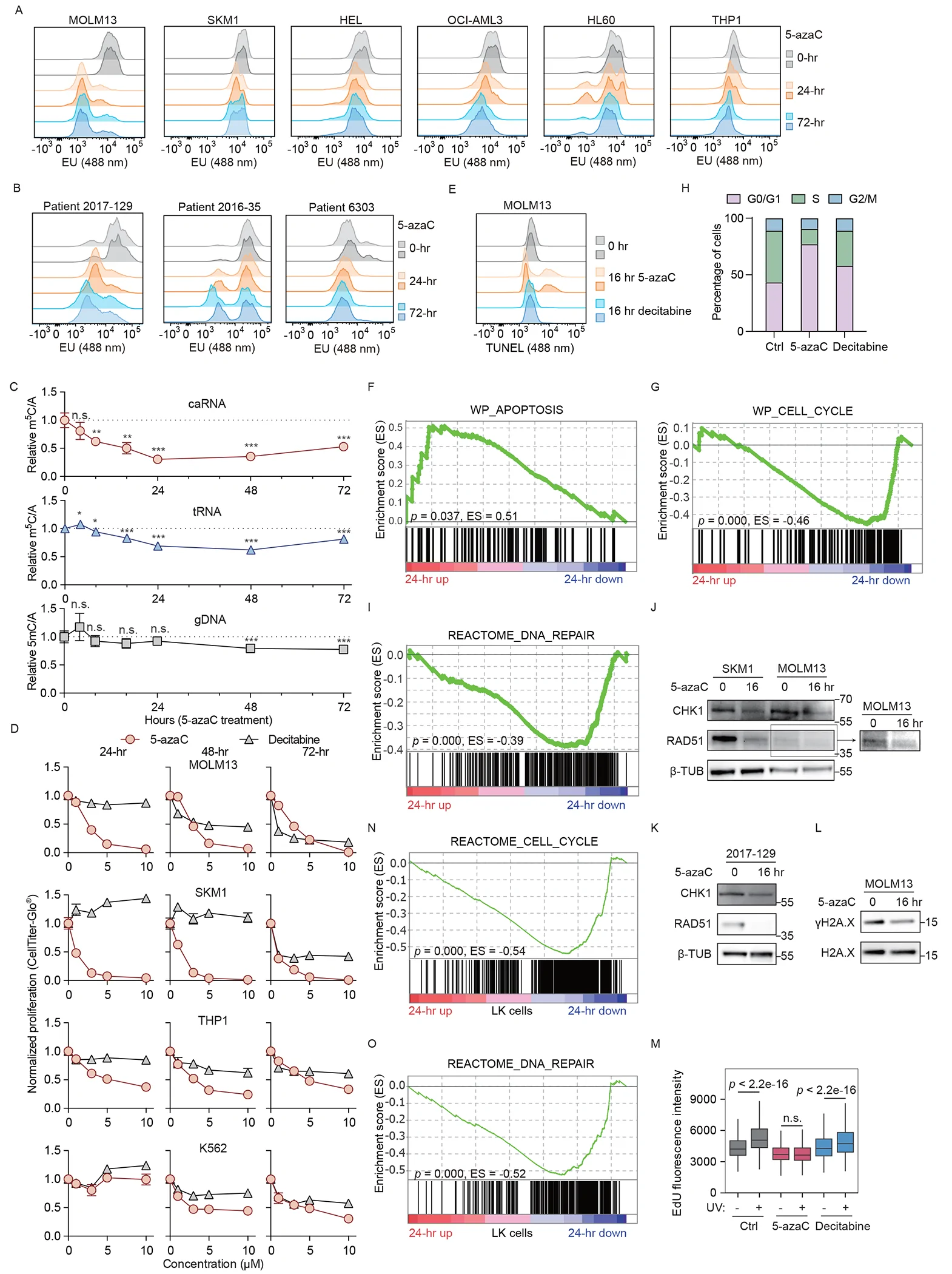
5-azaC induces RNA m^5^C depletion, transcriptional repression and leukemia cell death at early time points. (A and B) EU fluorescent signals in leukemia cell lines (A) and AML patient sample (B) after 5-azaC (3 μM, except for SKM1 with 1 μM) treatment. (C) Changes of m^5^C/A levels in caRNA and tRNA, and 5mC/A levels in gDNA after 5-azaC treatment in MOLM13. Modification levels are normalized to controls. (D) Cell proliferation of leukemia cell lines after 5-azaC or decitabine treatment. (E) TUNEL fluorescent signals in MOLM13 cells following 5-azaC or decitabine (3 μM) treatment. (F and G) GSEA of MSigDB apoptosis (F) and cell-cycle (G) gene set in 5-azaC-treated MOLM13. (H) Cell-cycle distribution of MOLM13 cells after a 16-h 5-azaC or decitabine (3 μM) treatment. (I) GSEA of MSigDB DNA repair gene set in 5-azaC-treated MOLM13. (J and K) Immunoblotting of DNA damage repair genes after 5-azaC treatment in SKM1, MOLM13 cells (J) and AML patient sample 2017-129 (K). (L) Immunoblotting of γH2A.X after 5-azaC treatment followed by 50J/m^2^ UVC irradiation. (M) EdU incorporation during DNA damage repair after UV irradiation in MOLM13 cells treated with 5-azaC or decitabine (3 μM) for 16 h. Only G1-phase populations are shown. (N and O) GSEA of MSigDB cell cycle (N) and DNA damage repair (O) gene set in 5-azaC-treated LK cells. Values in (C) and (D) represent the mean ± standard deviation (SD). All statistical significance was determined using an unpaired, two-sided Student’s *t* test. See also Figure S1 and S2, Table S2–S4.

**Figure 2.**
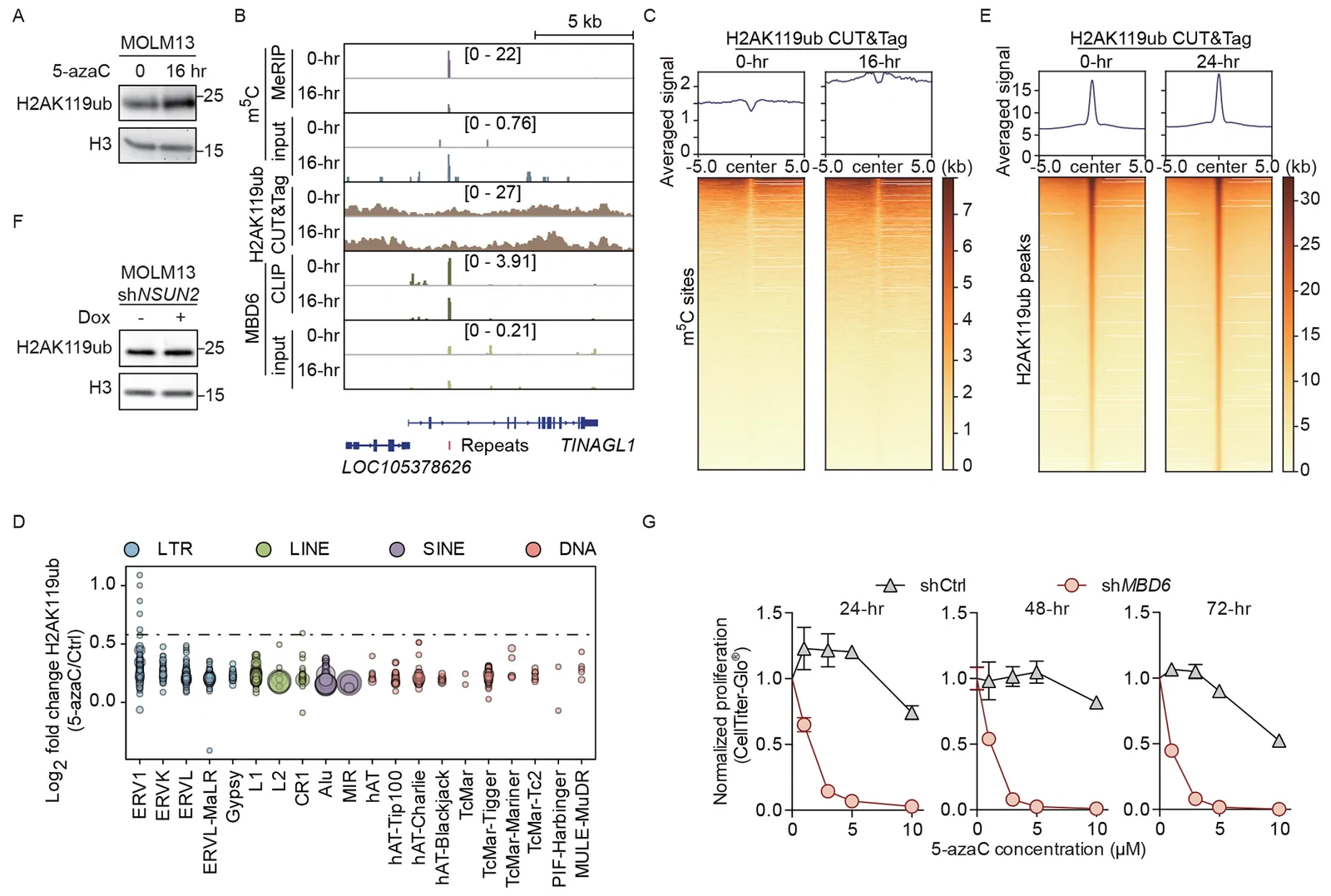
5-azaC induces transcriptional repression through m^5^C-dependent H2AK119ub regulation. (A) Immunoblotting of H2AK119ub changes after a 16-h 5-azaC treatment in MOLM13 cells. (B) Integrative Genome Viewer (IGV) tracks showing caRNA m^5^C MeRIP-seq, H2AK119ub CUT&Tag, and MBD6 CLIP-seq signals at the *TINAGL1* locus after a 16-h 5-azaC treatment. Track scales are indicated in brackets. (C) Metaplot and heatmap of H2AK119ub level changes at m^5^C sites after 5-azaC treatment in MOLM13 cells. (D) H2AK119ub changes across repeat families. Circle sizes represent normalized CUT&Tag read counts in control samples. The dashed line indicates a value of 0.58. (E) Metaplot and heatmap of H2AK119ub level changes at identified peaks after 5-azaC treatment in LK cells. (F) Immunoblotting of H2AK119ub changes following doxycycline (Dox)-induced NSUN2 depletion for 5 days in MOLM13 cells. (G) MOLM13 cell proliferation following 5-azaC treatment after 48-h shRNA-mediated MBD6 depletion. Time points indicate hours after 5-azaC treatment. The values represent mean ± SD. See also Figure S3 and S4.

**Figure 3.**
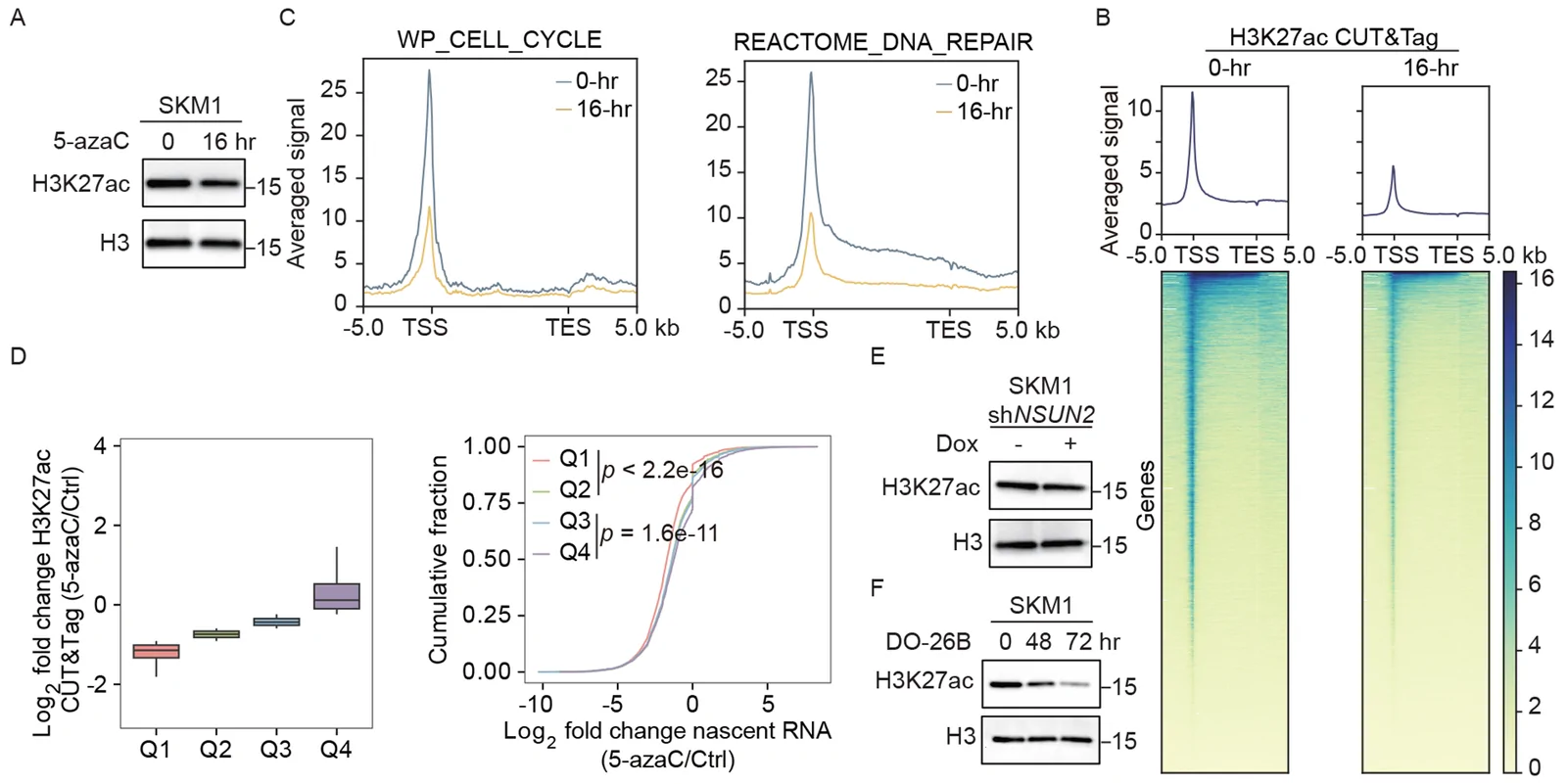
5-azaC induces transcriptional repression through H3K27ac downregulation. (A) Immunoblotting of H3K27ac changes in SKM1 cells after a 16-h 5-azaC treatment. (B) Metaplot and heatmap of H3K27ac changes across all annotated genes after a 16-h 5-azaC treatment in SKM1 cells. (C) Metaplot of H3K27ac changes in cell cycle and DNA damage repair gene sets in SKM1 cells. (D) H3K27ac (left) and nascent RNA-seq (right) changes in genes grouped into four quantiles based on fold changes of nearby H3K27ac peaks in SKM1 cells. Q1 represents the quantile with the most negative fold change. Statistical significance was determined using an unpaired, two-sided Student’s *t* test. (E and F) Immunoblotting of H3K27ac changes after a 5-day Dox-induced depletion of NSUN2 (E) or 5 μM DO-26B treatment (F) in SKM1 cells. See also Figure S5.

**Figure 4.**
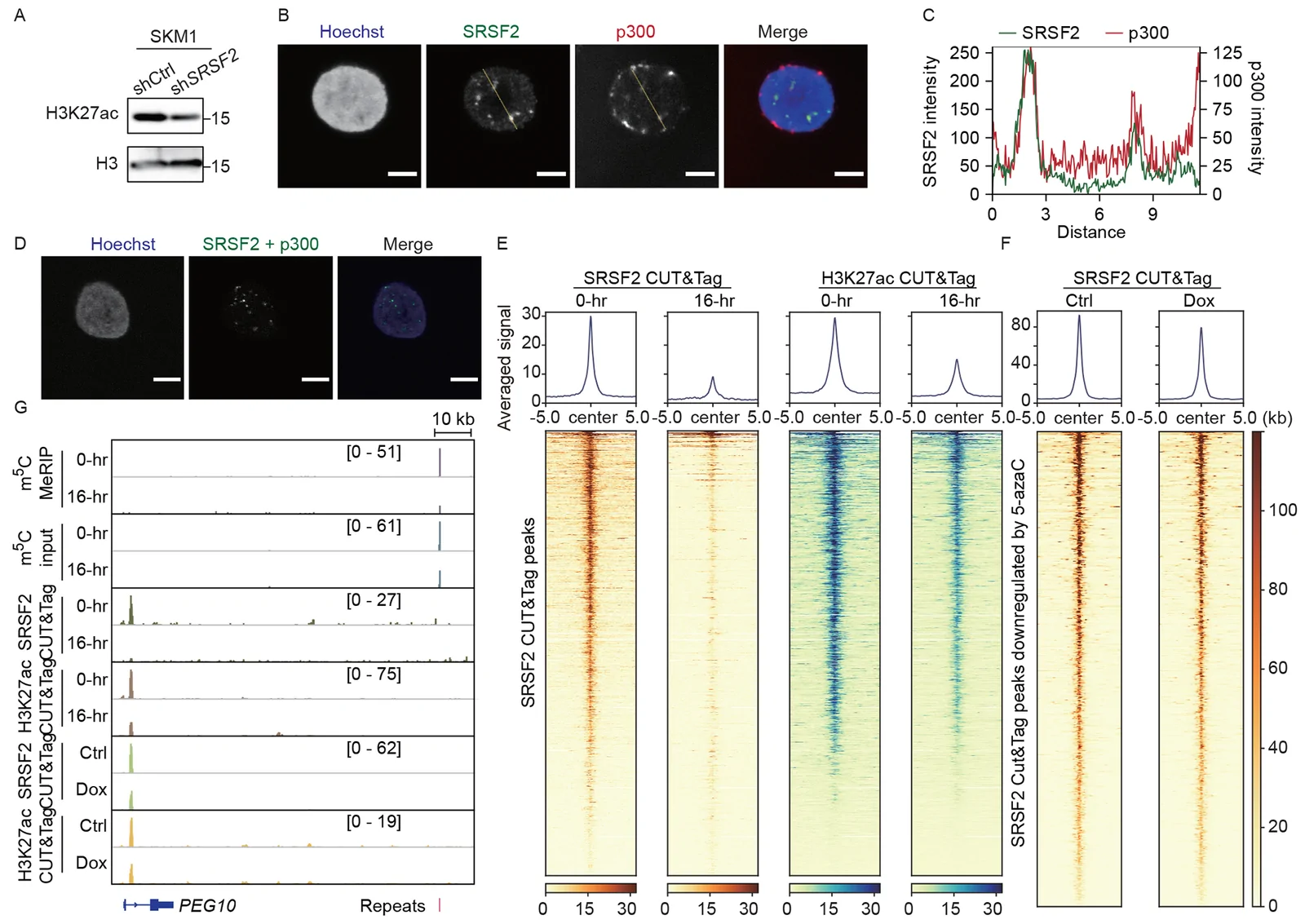
5-azaC downregulates H3K27ac through the SRSF2-mediated p300 recruitment. (A) Immunoblotting of H3K27ac changes after a 48-h shRNA-mediated SRSF2 depletion in SKM1 cells. (B) Co-immunofluorescence of SRSF2 and p300 in SKM1 cells. Scale bar: 5 μm. (C) Quantification of SRSF2-p300 colocalization along the yellow line in (B). (D) PLA of SRSF2 and p300 in SKM1 cells. Scale bar: 5 μm. (E) Metaplot and heatmap of SRSF2 and H3K27ac changes at SRSF2 target sites after 5-azaC treatment. (F) Metaplot and heatmap of SRSF2 CUT&Tag level changes at downregulated SRSF2 peaks after 5-azaC treatment. Samples were treated with Dox for 5-day NSUN2 depletion. (G) IGV tracks showing caRNA m^5^C MeRIP-seq, SRSF2 CUT&Tag, and H3K27ac CUT&Tag signals at genomic locus near *PEG10* after a 16-h 5-azaC treatment or Dox-induced NSUN2 depletion. Track scales are indicated in brackets. See also Figure S6.

**Figure 5.**
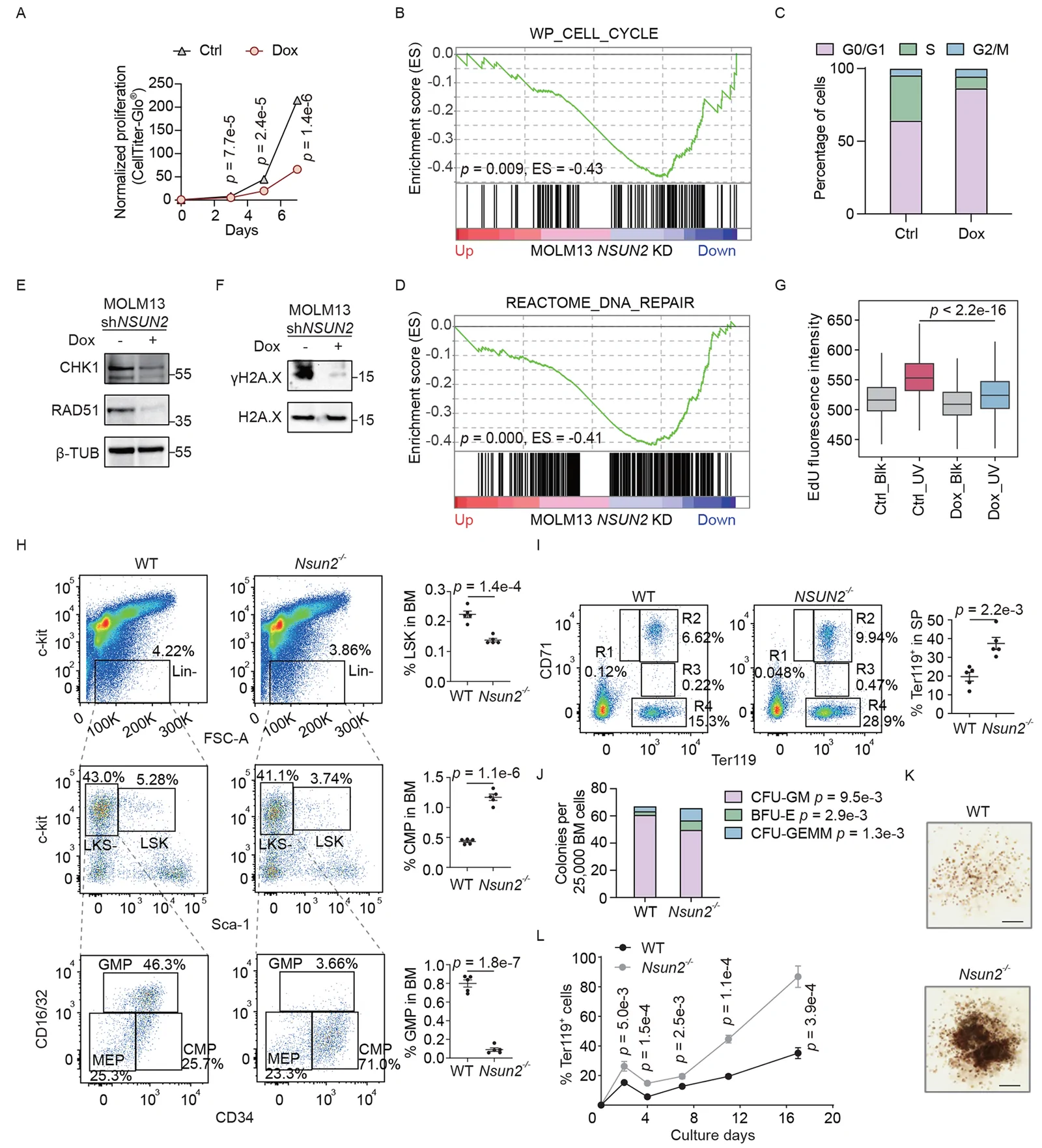
NSUN2 regulates leukemia proliferation and hematopoietic lineage commitment. (A) Cell proliferation of MOLM13 cells after Dox-induced NSUN2 depletion. (B) GSEA of MSigDB cell-cycle gene set in MOLM13 cells after a 5-day Dox-induced NSUN2 depletion. (C) Cell-cycle distribution of MOLM13 cells after a 5-day Dox-induced NSUN2 depletion. (D) GSEA of MSigDB DNA repair gene set in MOLM13 cells after a 5-day Dox-induced NSUN2 depletion. (E) Immunoblotting of DNA repair genes in MOLM13 cells after a 5-day Dox-induced NSUN2 depletion. (F) Immunoblotting of γH2A.X in MOLM13 cells after a 5-day Dox-induced NSUN2 depletion followed by 50 J/m^2^ UVC irradiation. (G) EdU incorporation during DNA damage repair after UVC irradiation in MOLM13 cells after a 5-day Dox-induced NSUN2 depletion. Only G0/G1-phase populations are shown. Blk, no irradiation. The box represents 25%, 50%, and 75% quantiles, while the whiskers extend to the most extreme data point that is within 1.5-fold IQR. (H) Flow cytometry analysis of LSK, LKS-, GMP, MEP, and CMP populations in BM from 6-month-old wild-type (WT) and *Nsun2*^−/−^ mice (left), and quantification of LSK, CMP and GMP populations in the total BM cells (right). N = 5 mice/genotype. (I) Flow cytometry analysis of erythroid cells in the spleen of 6-month-old WT and *Nsun2*^−/−^ mice (left), and quantification of the Ter119^+^ erythroblasts (right). R1: pro-, R2: basophilic-, R3: late basophilic- and polychromatophilic-, and R4: orthochromatophilic-erythroblasts. SP, spleen. (J) CFU-C assay of BM cells from 6-month-old WT and *Nsun2*^−/−^ mice. (K) Representative GEMM colonies formed by BM cells from 6-month-old WT and *Nsun2*^−/−^ mice. Scale bar, 200 μm. (L) Quantification of Ter119*+* erythroid cells percentage at different time points during *in vitro* erythroid differentiation using the hematopoietic stem and progenitor LK cells derived from mouse BM cells (n = 3). Statistical significance was determined using an unpaired, two-sided Student’s t test. See also Figure S7 and Table S5.

**Figure 6.**
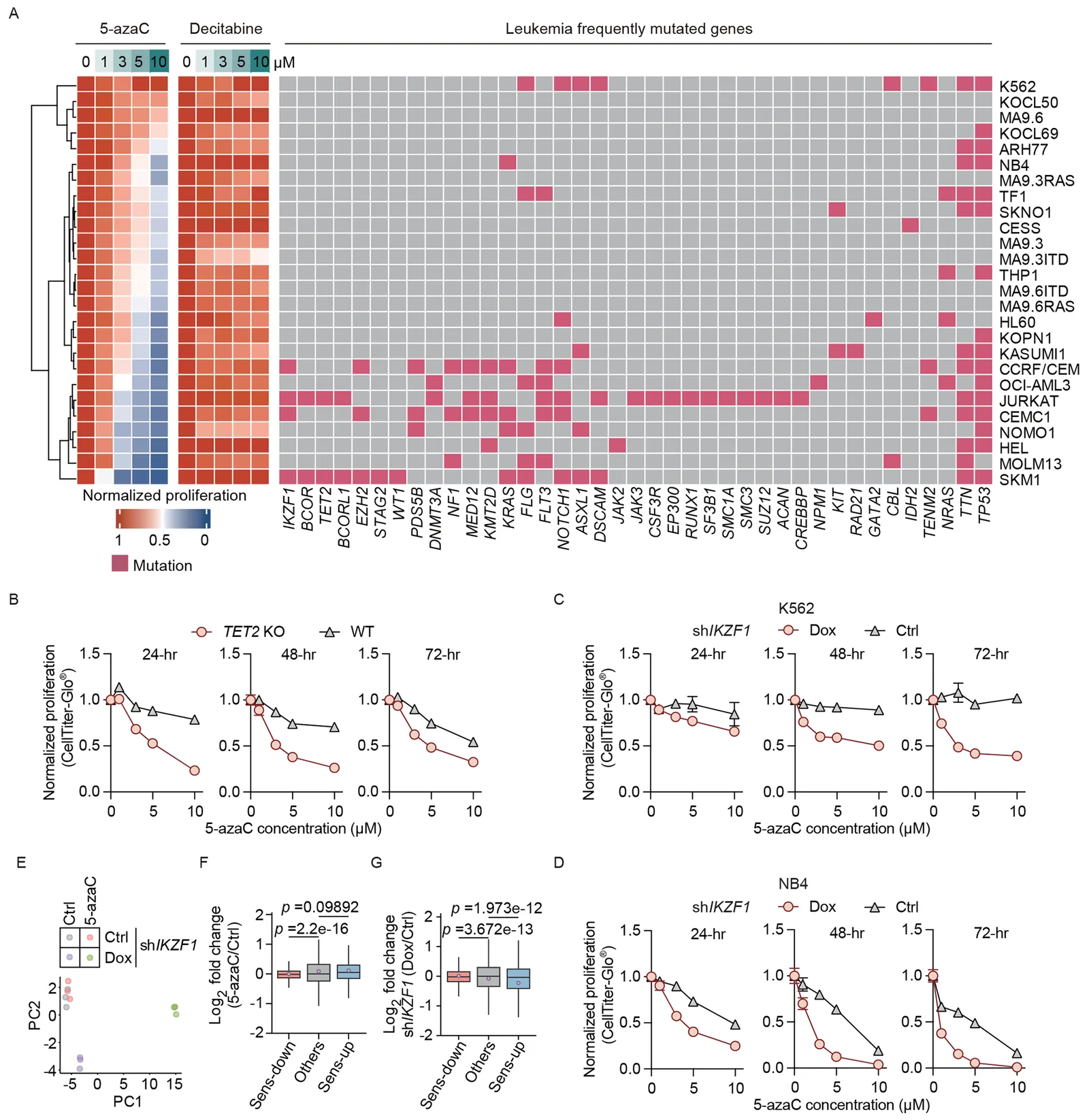
Screening of leukemia cell lines infers mutations associated with 5-azaC sensitivity. (A) Proliferation of 26 leukemia cell lines after a 24-h treatment with 5-azaC (left) or decitabine (middle), along with their reported mutations (right) from public databases (DepMap: 24Q2; COSMIC: v100; Cellosaurus: release 47). Proliferation was measured by CellTiter-Glo^®^. (B) Proliferation of THP1 WT and *TET2* KO cells after 5-azaC treatment. (C and D) Proliferation of K562 (C) and NB4 (D) cells treated with 5-azaC after Dox-induced IKZF1 depletion. The denoted time indicates hours after 5-azaC treatment. (E) PCA of RNA-seq of K562 cells with Dox-induced IKZF1 depletion and/or a 24-h 5-azaC treatment (F and G) Differential expression of Sens-up, Sens-down and other genes (Others) in K562 cells with 5-azaC treatment without IKZF1 depletion (F) or Dox-induced IKZF1 depletion without 5-azaC treatment (G). The lilac circle represents value of mean in each group of genes. All values represent the mean ± SD. Statitical significance was determined using an unpaired, two-sided Student’s t test. See also Figure S8.

**Table:** KEY RESOURCES TABLE

REAGENT OR RESOURCE	SOURCE	IDENTIFIER
Antibodies		
APC-anti-Lineage cocktail	BD Biosciences	Cat# 5508074
PE-Cy^™^7-anti-Sca1	BD Biosciences	Cat# 558162; RRID: AB_647253
PE-anti-c-kit	BD Biosciences	Cat# 553869; RRID: AB_395103
Alexa Flour 700-anti-CD34	BD Biosciences	Cat# 560518; RRID: AB_1727471
APC-Cy7-anti-CD16/32	BioLegend	Cat# 101327; RRID: AB_1967102
BV421-anti-CD135	BD Biosciences	Cat# 562898; RRID: AB_2737876
PerCP-Cy^™^5.5-anti-Gr-1	BD Biosciences	Cat# 552093; RRID: AB_394334
PE-anti-CD11b (Mac-1)	BD Biosciences	Cat# 553311; RRID: AB_394775
APC-anti-Ter119	BD Biosciences	Cat# 557909; RRID: AB_398635
PE-anti-CD71	BD Biosciences	Cat# 553267; RRID: AB_394744
anti-CHK1	Cell Signaling Technology	Cat# 2360S; RRID: AB_2080320
anti-RAD51	Cell Signaling Technology	Cat# 8875S; RRID: AB_2721109
anti-H3	Cell Signaling Technology	Cat# 12648S; RRID: AB_2797978
anti-H2A.X	Cell Signaling Technology	Cat# 2595S; RRID: AB_10694556
anti-γH2A.X	Cell Signaling Technology	Cat# 9718S; RRID: AB_2118009
anti-H2AK119ub	Cell Signaling Technology	Cat# 8240S; RRID: AB_10891618
anti-H3K27ac	Abcam	Cat# ab4729; RRID: AB_2118291
anti-β-TUB	Proteintech	Cat# HRP-66240; RRID: AB_2883838
anti-SRSF2	Abcam	Cat# ab204916; RRID: AB_2909393
anti-Myc-tag	Cell Signaling Technology	Cat# 2276S; RRID: AB_331783
anti-rabbit IgG	Cell Signaling Technology	Cat# 7074S; RRID: AB_2099233
anti-mouse IgG	Cell Signaling Technology	Cat# 7076S; RRID: AB_330924
anti-NSUN2	Proteintech	Cat# 20854-1-AP; RRID: AB_10693629
anti-SRSF2	Abcam	Cat# ab11826; RRID: AB_298608
anti-p300	Cell Signaling Technology	Cat# 86377S; RRID: AB_2800-77
goat anti-mouse IgG-AF 546	Thermo Scientific	Cat# A-11030; RRID: AB_2737024
goat anti-rabbit IgG-AF 647	Thermo Scientific	Cat# A-21244; RRID: AB_2535812
anti-SRSF2	Proteintech	Cat# 20371-1-AP; RRID: AB_2616311
anti-SRSF2	Sigma-Aldrich	Cat# S4045; RRID: AB_477511
Rabbit IgG	Cell Signaling Technology	Cat# 2729S; RRID: AB_1031062
Mouse (G3A1) mAb IgG1 Isotype Control	Cell Signaling Technology	Cat# 5415S; RRID: AB_10829607
anti-m^5^C	Diagenode	Cat# MAb-081-100
anti-H3K27ac	Active motif	Cat# 39034
PerCP-Cy^™^5.5-anti-CD45.2	BD Biosciences	Cat# 552950; RRID: AB_394528
FITC-anti-CD45.1	BD Biosciences	Cat# 553775; RRID: AB_395043
PE-Cy^™^7-anti-Gr-1	BD Biosciences	Cat# 565033; RRID: AB_2739049
APC-anti-B220	BD Biosciences	Cat# 553092; RRID: AB_398531
PE-anti-CD8a	BD Biosciences	Cat# 553033; RRID: AB_394571
PE-Cy^™^7-anti-CD4	BD Biosciences	Cat# 552775; RRID: AB_394461
APC-Cy7-anti-IgM	BioLegend	Cat# 406516
anti-MBD6	Dr. Lu Wang	N/A
Bacterial and virus strains		
NEB^®^ Stable Competent *E. coli* (High Efficiency)	NEB	Cat# C3040H
Biological samples		
See Table S3	N/A	N/A
Chemicals, peptides, and recombinant proteins		
RPMI1640 medium	Gibco	Cat# 22400089
DMEM (high glucose, pyruvate)	Gibco	Cat# 11995073
FBS	Gibco	Cat# 2614079
Penicillin-Streptomycin	Gibco	Cat# 15140122
Recombinant human GM-CSF	PeproTech	Cat# 300-03
IMDM	Gibco	Cat# 12440053
Recombinant human SCF	PeproTech	Cat# 300-07
Recombinant human TPO	PeproTech	Cat# 300-18
Recombinant human FLT3L	PeproTech	Cat# 300-19
Recombinant human IL-3	PeproTech	Cat# 200-03
Recombinant human IL-6	PeproTech	Cat# 200-06
TransDux MAX Lentivirus Transduction Reagent	System Biosciences	Cat# LV860A-1
Lipofectamine^™^ LTX Reagent with PLUS^™^ Reagent	Invitrogen	Cat# 15338100
Opti-MEM^™^ I Reduced Serum Medium	Gibco	Cat# 31985070
StemSpan^™^ SFEMII	Stem Cell Technologies	Cat# 09655
Recombinant mouse TPO	PeproTech	Cat# 315-14
Recombinant mouse FLT3 Ligand	PeproTech	Cat# 300-19
StemRegenin 1	Stem Cell Technologies	Cat# 72354
Methylcellulose medium	MethoCult	Cat# M3134
Recombinant mouse SCF	PeproTech	Cat# 250-03
Recombinant mouse IL-3	PeproTech	Cat# 213-13
Recombinant mouse GM-CSF	PeproTech	Cat# 315-03
Recombinant human EPO	PeproTech	Cat# 100-64
Schneider’s *Drosophila*-Medium	Gibco	Cat# 21720024
NuPAGE^™^ LDS sample buffer	Invitrogen	Cat# NP0007
Proteinase inhibitor cocktail	Sigma-Aldrich	Cat# P8340
BSA	Fisher Scientific	Cat# BP1600-1
PBST	Pierce	Cat# PI28352
proteinase K	Thermo Scientific	Cat# EO0491
TURBO DNase^™^	Invitrogen	Cat# AM2239
TRIzol^™^ reagent	Invitrogen	Cat# 15596026
Nuclease P1	NEB	Cat# M0660S
Shrimp alkaline phosphatase	NEB	Cat# M0371S
Phosphodiesterase I	Sigma-Aldrich	Cat# P3243
5-Ethynyl-2′-deoxyuridine	Sigma-Aldrich	Cat# 900584
5-Fluoro-2’-deoxyuridine	Sigma-Aldrich	Cat# 343333
Thymidine	Sigma-Aldrich	Cat# T1895
Hoechst 33342	Abcam	Cat# ab228551
ProLong^™^ Glass Antifade Mountant	Invitrogen	Cat# P36980
Laemmli sample buffer	Bio-Rad	Cat# 1610747
RNaseOUT^™^ Recombinant Ribonuclease Inhibitor	Invitrogen	Cat# 10777019
SUPERase·In^™^ RNase Inhibitor	Invitrogen	Cat# AM2696
NEBNext^®^ Magnesium RNA Fragmentation buffer	NEB	Cat# E6186A
buffer RLT	Qiagen	Cat# 79216
RNase T1	Thermo Scientific	Cat# EN0642
T4 PNK	NEB	Cat# M0201L
T4 RNA ligase 1	NEB	Cat# M0437M
Critical commercial assays		
SF cell line 4D-nucleofector kit	Lonza	Cat# V4XC-2032
CellTiter-Glo^®^ 2.0 Cell Viability Assay	Promega	Cat# G9242
SuperSignal^™^ West Dura Extended Duration Substrate kit	Thermo Scientific	Cat# 34075
RNA Clean & Concentrator-25 kit	Zymo Research	Cat# R1017
PureLink^™^ Genomic DNA Mini Kit	Invitrogen	Cat# K182001
DeadEnd Fluorometric TUNEL System	Promega	Cat# G3250
Click-iT^™^ RNA Alexa Fluor^™^ 488 Imaging Kit	Invitrogen	Cat# C10329
Duolink^®^ Proximity Ligation Assay kit	Millipore	Cat# DUO92002, DUO92004, DUO92008
Dynabeads^™^ Protein G	Invitrogen	Cat# 1003D
Dynabeads^™^ Protein A	Invitrogen	Cat# 1001D
ERCC RNA spike-in mix	Invitrogen	Cat# 4456740
Pierce^™^ IP lysis buffer	Thermo Scientific	Cat# 87787
SMARTer^®^ Stranded Total RNA-Seq Kit v2 - Pico Input Mammalian	Takara	Cat# 634412
Click-iT^™^ Nascent RNA Capture Kit for gene expression analysis	Invitrogen	Cat# C10365
RiboMinus Eukaryote System v2	Invitrogen	Cat# A15026
CUT&Tag-IT Assay Kit	Active motif	Cat# 53160
CUT&Tag-IT^®^ Spike-In Control	Active motif	Cat# 53168
NEBNext^®^ Small RNA Library Prep Set	NEB	Cat# E7330S
Deposited data		
Next generation sequencing of RNA	This study	GEO: GSE297591
CUT&Tag	This study	GEO: GSE297589
Experimental models: Cell lines		
MA9.3	Dr. Jianjun Chen	N/A
MA9.3ITD	Dr. Jianjun Chen	N/A
MA9.3RAS	Dr. Jianjun Chen	N/A
MA9.6	Dr. Jianjun Chen	N/A
MA9.6ITD	Dr. Jianjun Chen	N/A
MA9.6RAS	Dr. Jianjun Chen	N/A
KOPN1	Dr. Jianjun Chen	RRID: CVCL_3937
JURKAT	Dr. Jianjun Chen	RRID: CVCL_0065
KOCL50	Dr. Jianjun Chen	RRID: CVCL_6866
TF1	Dr. Jianjun Chen	RRID: CVCL_0559
SKNO1	Dr. Jianjun Chen	RRID: CVCL_2196
HEL	Dr. Jianjun Chen	RRID: CVCL_0001
KOCL69	Dr. Jianjun Chen	RRID: CVCL_3995
NB4	Dr. Jianjun Chen	RRID: CVCL_0005
K562	ATCC	Cat# CCL-243; RRID: CVCL_0004
MOLM13	DSMZ	Cat# ACC554; RRID: CVCL_2119
OCI-AML3	DSMZ	Cat# ACC582; RRID: CVCL_1844
SKM1	DSMZ	Cat# ACC547; RRID: CVCL_0098
NOMO1	DSMZ	Cat# ACC542; RRID: CVCL_1609
CEM/C1	ATCC	Cat# CRL-2265; RRID: CVCL_3496
CCRF/CEM	ATCC	Cat# CCL-119; RRID: CVCL_0207
HL60	ATCC	Cat# CCL-240; RRID: CVCL_0002
THP1	ATCC	Cat# TIB-202; RRID: CVCL_0006
CESS	ATCC	Cat# TIB-190; RRID: CVCL_0209
ARH77	ATCC	Cat# CRL-1621; RRID: CVCL_1072
KASUMI1	ATCC	Cat# CRL-2724; RRID: CVCL_0589
HEK293T	ATCC	Cat# CRL-3216; RRID: CVCL_0063
*Drosophila* S2 cells	Gibco	Cat# R69007; RRID: CVCL_Z232
Experimental models: Organisms/strains		
Mouse: global *Nsun2^−/−^*	Dr. Michaela Frye	N/A
Oligonucleotides		
See Table S1	N/A	N/A
Recombinant DNA		
pCMV-VSV-G	Addgene	Cat# 8454; RRID:Addgene_8454
pCMV-dR8.2	Addgene	Cat# 8455; RRID:Addgene_8455
Tet-pLKO-puro	Addgene	Cat# 21915; RRID:Addgene_21915
Software and algorithms		
Prism 10	GraphPad	https://www.graphpad.com/
ImageJ v2.0.0	Rasband	https://imagej.net/ij/
IGV (Integrative Genomics Viewer) v2.13.1	Broad Institute	https://igv.org/
RStudio v4.3.3	Posit PBC	https://posit.co/products/open-source/rstudio/
Flowjo Software v10.9.0	Flowjo	https://www.flowjo.com/
DESeq2 v1.42.1	Love et al.^80^	https://bioconductor.org/packages/release/bioc/html/DESeq2.html
RUVSeq v1.36.0	Risso et al.^81^	https://bioconductor.org/packages/release/bioc/html/RUVSeq.html
Cutadapt v4.8	Martin et al.^82^	https://cutadapt.readthedocs.io/en/stable/
HISAT2 v2.2.1	Kim et al.^83^	https://daehwankimlab.github.io/hisat2/
bowtie2 v2.4.4	Langmead and Salzberg^84^	https://bowtie-bio.sourceforge.net/bowtie2/index.shtml
featureCounts v2.0.3	Liao et al.^85^	https://subread.sourceforge.net/featureCounts.html
GSEA v4.3.3	Subramanian et al.^86^	https://www.gsea-msigdb.org/gsea/index.jsp
RepEnrich2	Criscione et al.^87^	https://github.com/nerettilab/RepEnrich2
MACS3 v3.0.1	Zhang et al.^88^	https://github.com/macs3-project/MACS
DeepTools v3.5.5	Ramirez et al.^89^	https://deeptools.readthedocs.io/en/develop/#
Picard v2.22.8	Broad Institute	https://broadinstitute.github.io/picard/
DiffBind v3.12.0	Stark and Brown	https://bioconductor.org/packages/release/bioc/html/DiffBind.html
Homer v4.11.1	Heinz et al.^90^	http://homer.ucsd.edu/homer/
SJARACNe v0.2.1	Khatamian et al.^91^	https://github.com/jyyulab/SJARACNe
NetBID2.0	Dong et al.^72^	https://jyyulab.github.io/NetBID/
Other		
		

## Data Availability

Sequencing data generated from this study have been deposited in the Gene Expression Omnibus (GEO) and made accessible under accession numbers GEO: GSE297589 and GSE297591 This paper does not report original code. Any additional information required to reanalyze the data reported in this paper is available from the lead contact upon request.
