# Supplementary material for: 5-azacytosine induces cytotoxicity via 5-methylcytosine depletion on chromatin-associated RNA in leukemia
Source: Mol Cell. Author manuscript; Available in PMC 2026 Oct 27. (PMC13711426; doi:10.1016/j.molcel.2026.08.018)
Supplement: Supplementary file 1 [file NIHMS2215459-supplement-SupFile_5azaC.docx]

**Supplemental information**

**5-azacytosine induces cytotoxicity via 5-methylcytosine depletion on chromatin-associated RNA in leukemia**

Boyang Gao, Ying Li, Xinyuan Ma, Long Zhao, Zhongyu Zou, Juyeong Hong, Junhong Xiang, Xiaoyang Dou, Feng-Chun Yang, Mingjiang Xu, Chuan He

Figure S1. 5-azaC induces global transcriptional repression. Related to Figure 1.

(A) Schematics of the metabolic pathways of 5-azaC and decitabine.

(B) Normalized median of the EU fluorescent signals in leukemia cells and AML patient sample after 5-azaC treatment.

(C and D) Nascent RNA-seq of MOLM13 (C) and SKM1 (D) cells after 5-azaC (3 μM) treatment. DEGs are defined as |log_2_ fold change (5-azaC/Ctrl) | > 1 and adj.*p*-value < 0.05. DEG numbers are annotated on the plot. Adj.*p*-value, adjusted *p*-value.

(E) RNA electroporation by TapeStation of aza-IP eluates from MOLM13 cells treated with 3 μM 5-azaC for the indicated durations. RNA size markers are annotated on the left (bp).

(F) EU fluorescent signals in MOLM13 after longer 5-azaC (3 μM) treatment.

Figure S2. 5-azaC modulates cell cycle, DNA damage repair, and apoptosis mediated by RNA m^5^C. Related to Figure 1.

(A and B) Volcano plot of RNA-seq from MOLM13 cells (A) and SKM1 cells (B) after a 24-hour 5-azaC (3 μM) treatment. DEGs are defined as |log_2_ fold change| > 0.58 and adj.*p*-value < 0.05. DEG numbers are annotated on the plot. Adj.*p*-value, adjusted *p*-value.

(C and D) GO analysis of the top enriched biological processes in DEGs after 5-azaC (3 μM) treatment in MOLM13 (C) and SKM1 (D) cells. The top 20 GO terms enriched in downregulated genes and the top 9 GO terms enriched in upregulated genes with FDR < 0.05 were plotted.

(E and F) GSEA of transcriptional changes in cell cycle gene set based on nascent RNA-seq in 5-azaC-treated (3 μM) MOLM13 (E) and SKM1 (F).

(G and H) Cell cycle distribution of SKM1 cells (G) and AML patient sample 2017-129 (H) after 16 hours 5-azaC or decitabine (3 μM) treatment.

(I and J) GSEA of transcriptional changes in DNA repair gene set based on nascent RNA-seq in 5-azaC-treated (3 μM) MOLM13 (I) and SKM1 (J).

(K) EdU incorporation during DNA damage repair after UVC irradiation in MOLM13 cells treated with 3 μM of 5-azaC or decitabine for 16 hours. Only G2/M-phase populations are shown.

(L and M) EdU incorporation during DNA damage repair after UV irradiation in MOLM13 cells treated with 3 μM of 5-azaC for 16 hours. ActD was added to the media at 1 μg/mL for 2 hours prior to irradiation. Only G0/G1- (L) and G2/M- (M) phase populations are shown.

(N) Cell proliferation of MOLM13 after 3 μM of 5-azaC or decitabine treatment in combination with 0.2 nM venetoclax for 24 hours.

**Figure S3. 5-azaC induces caRNA m^5^C depletion. Related to Figure 2.**

(A and B) Metaplot of caRNA m^5^C MeRIP-seq signal changes at m^5^C sites from 5-azaC (3 μM) treated and non-treated MOLM13 (A) and SKM1 (B) cells.

(C and D) Percentage of sequencing reads mapped to repetitive elements (Repeats) or not (Others) in caRNA input and MeRIP samples from control MOLM13 (C) and SKM1 (D) cells. The percentage is the average of three replicates.

(E) m^5^C enrichment across repeat families in caRNA from control MOLM13 cells. Circle sizes represent normalized read counts from MeRIP-seq. The dashed line indicates a value of 1.

(F) Differential m^5^C enrichment across repeat families in MOLM13 caRNA after 16 hours of 5-azaC (3 μM) treatment. Circle sizes represent normalized MeRIP-seq read counts in untreated samples. The dashed line indicates a value of -0.58.

(G) Differential levels of caRNA repeat subfamilies in MOLM13 cells after a 16-hour 5-azaC (3 μM) treatment. Circle sizes represent normalized caRNA levels in untreated samples.

(H) Differential levels of caRNA transcripts from genes adjacent to m^5^C sites (m^5^C) versus other genes (Others).

**Figure S4. 5-azaC induces transcriptional repression through m^5^C-dependent H2AK119ub regulation. Related to Figure 2.**

(A) Immunoblotting of H2AK119ub changes following 5-azaC treatment in 2017-129 sample.

(B and C) Metaplot of H2AK119ub changes across all annotated genes (B) or specific gene sets (C) after a 16-hour 5-azaC (3 μM) treatment in MOLM13 cells.

(D) Metaplot and heatmap of MBD6 CLIP-seq signal changes at caRNA m^5^C sites that are downregulated after a 16-hour 5-azaC (3 μM) treatment in MOLM13 cells.

(E) KD efficiency of m^5^C methyltransferases or *DNMT1* after a 5-day Dox-induced depletion of the respective gene. Expression levels were first normalized to *ACTB*, followed by normalization to the respective controls.

(F) Immunoblotting of H2AK119ub changes following a Dox-induced depletion of m^5^C methyltransferases or *DNMT1* for 5 days in MOLM13 cells.

(G) Cell cycle distribution measured by EdU incorporation in MOLM13 cells following 48-hour shRNA-mediated MBD6 depletion, followed by a 16-hour 5-azaC (3 μM) treatment.

**Figure S5. 5-azaC induces transcriptional repression through H3K27ac downregulation. Related to Figure 3.**

(A) Immunoblotting of H3K27ac changes in MOLM13 cells (upper) and AML patient sample 2017-129 (lower) treated with 3 μM of 5-azaC.

(B) Immunoblotting of H3K27ac changes in SKM1 cells treated with 1 μM of 5-azaC replenished every 24 hours.

(C) Metaplot and heatmap of H3K27ac level changes across all annotated genes after a 16-hour 5-azaC (3 μM) treatment in MOLM13 cells.

(D) Metaplot of H3K27ac changes in cell cycle (upper) and DNA damage repair (lower) gene sets in MOLM13 cells.

(E) H3K27ac (left) and nascent RNA-seq (right) changes in genes grouped into four quantiles based on fold changes of nearby H3K27ac peaks in MOLM13 cells. Q1 represents the quantile with the most negative fold change.

(F and G) H3K27ac changes across repeat families in SKM1 (F) and MOLM13 (G) cells. Circle sizes represent normalized CUT&Tag read counts in control samples. The dashed line indicates a value of -0.58.

(H and I) Metaplot of H3K27ac level changes at caRNA m^5^C sites from the corresponding cell lines after 5-azaC (3 μM) treatment in SKM1 (H) and MOLM13 (I) cells.

(J) Immunoblotting of H3K27ac changes following a Dox-induced depletion of RNA m^5^C methyltransferases or DNMT1 in MOLM13 cells.

(K) Metaplot of H3K27ac level changes at caRNA m^5^C sites after a 5-day Dox-induced NSUN2 depletion in SKM1 cells.

**Figure S6. 5-azaC downregulates H3K27ac through the SRSF2-mediated p300 recruitment. Related to Figure 4.**

(A) Immunoblotting of equal proportions of nucleoplasm (Nuc) and chromatin (Chr) fractions from SKM1 cells. The images were cropped from the same original image with no modification of intensity.

(B) KD efficiency of *SRSF2* after a 48-hour shRNA-mediated depletion. Expression levels were first normalized to *ACTB*, followed by normalization to the respective controls.

(C) Immunoblotting of H3K27ac changes after a 48-hour shRNA-mediated SRSF2 depletion in MOLM13 cells.

(D) Immunoblotting of SRSF2 levels across different cell lines. The samples were loaded with the same cell number.

(E) Immunoprecipitation of SRSF2 with different antibodies: Abc (SRSF2 antibody ab204916), Ptg (SRSF2 antibody 20371-1-AP), Sig (SRSF2 antibody S4045).

(F) Immunoblotting of SRSF2-Myc overexpression in K562 cells (upper) and Myc-tag CLIP followed by end ligation of pCp-AF488 fluorophore (lower).

(G) Metaplot of SRSF2 CUT&Tag signal changes in cell cycle (upper) and DNA repair (lower) gene sets in SKM1 cells.

(H) Metaplot and heatmap of SRSF2 CUT&Tag signal changes at caRNA m^5^C sites after a 16-hour 5-azaC (3 μM) treatment in SKM1 cells.

(I and J) Metaplot of SRSF2 CUT&Tag signal changes across all annotated genes (I) or specific gene sets (J) following a 5-day Dox-induced NSUN2 depletion in SKM1 cells.

**Figure S7. NSUN2 regulates leukemia proliferation and hematopoietic lineage commitment. Related to Figure 5.**

(A) Cell cycle distribution of SKM1 cells after a 5-day Dox-induced NSUN2 depletion.

(B) EdU incorporation during DNA damage repair after UVC irradiation in MOLM13 cells after a 5-day Dox-induced NSUN2 depletion. Only G2/M-phase populations are shown. Blk, no irradiation.

(C) Cell proliferation of HEL with *NSUN2* overexpression followed by a 24-hour 5-azaC treatment.

(D) KD efficiency of *Nsun2*^-/-^ mice measured in LK cells.

(E and F) Flow cytometry analysis of LK (E) and erythroid (F) cell populations in progeny cells from WT and *Nsun2*^-/-^ CFU-C assays shown in Figure 5J.

(G) Quantification of CD45.2^+^ cells in the PB of recipient mice at different time points after transplantation.

(H) Quantification of CD45.2^+^ cells in the BM of recipient mice at 32 weeks after transplantation.

(I) Quantification of donor-derived GMP population in the BM of recipient mice at 32 weeks after transplantation.

(J and K) Percentages of donor-derived lineage population in the PB (J) and BM (K) of recipient mice at 32 weeks after transplantation, including myeloid (Gr-1^+^/CD11b^+^) cells, erythroid (Ter119^+^) cells, T (CD4^+^ and CD8^+^) cells and B (B220^+^) cells.

(L-N) Flow cytometry analyses of Ter119^+^ erythroid cells at days 2 (L), 4 (M) and 17 (N) during *in vitro* erythroid differentiation (upper left), quantification of Ter119^+^ erythroid cells (upper right), and the representative images of the cell morphology stained by Wright-Giemsa following cytospin (lower). Scale bar, 20 µm.

**Figure S8. Gene network inference identifies mutations associated with 5-azaC sensitivity. Related to Figure 6.**

(A) KD efficiency of *IKZF1* and *NPM1* after a 5-day Dox-induced depletion of the respective gene. Expression levels were first normalized to *ACTB*, followed by normalization to the respective controls.

(B and C) Cell cycle distribution measured by EdU incorporation in K562 (B) or NB4 (C) cells following 5-day Dox-induced IKZF1 depletion, followed by a 16-hour 5-azaC (3 μM) treatment.

(D) Metaplot of IKZF1 ChIP-seq in K562 cells at caRNA m^5^C sites.

(E) Schematic representation of the identification of genes determining 5-azaC sensitivity through gene network-based inference using NetBID2.

(F) Expressions of frequently mutated genes in leukemia that correlate with 5-azaC sensitivity and their target genes in the network.

(G) GSEA of positive and negative targets of frequently mutated genes in leukemia related to 5-azaC sensitivity. Log_2_FC refers to log_2_ fold change gene expression (resistant/sensitive).

(H) Cell proliferation of THP1 following a 24-hour 5-azaC treatment after Dox-induced NPM1 depletion.

(I) Cell proliferation of HEL following a 24-hour 5-azaC treatment after 48-hour 10 μM tazemotostat treatment.
